# Regulating the Solvation Structure of Electrolyte via Dual–Salt Combination for Stable Potassium Metal Batteries

**DOI:** 10.1002/advs.202301201

**Published:** 2023-04-17

**Authors:** Jimin Park, Gwangeon Oh, Un‐Hyuck Kim, Muhammad Hilmy Alfaruqi, Xieyu Xu, Yangyang Liu, Shizhao Xiong, Adi Tiara Zikri, Yang‐Kook Sun, Jaekook Kim, Jang‐Yeon Hwang

**Affiliations:** ^1^ Department of Energy Engineering Hanyang University Seoul 06407 Republic of Korea; ^2^ Department of Materials Science & Engineering Chonnam National University Gwangju 61186 Republic of Korea; ^3^ State Key Laboratory for Mechanical Behavior of Materials Xi'an Jiaotong University 28 Xianning West Road Xi'an 710049 China; ^4^ Department of Physics Chalmers University of Technology Göteborg SE 412 96 Sweden

**Keywords:** electrolyte modulation, phase‐field modeling, potassium metal anode, potassium metal batteries

## Abstract

Batteries using potassium metal (K‐metal) anode are considered a new type of low‐cost and high‐energy storage device. However, the thermodynamic instability of the K‐metal anode in organic electrolyte solutions causes uncontrolled dendritic growth and parasitic reactions, leading to rapid capacity loss and low Coulombic efficiency of K‐metal batteries. Herein, an advanced electrolyte comprising 1 M potassium bis(fluorosulfonyl)imide (KFSI) + 0.05 M potassium hexafluorophosphate (KPF_6_) dissolved in dimethoxyethane (DME) is introduced as a simple and effective strategy of regulated solvation chemistry, showing an enhanced interfacial stability of the K‐metal anode. Incorporating 0.05 M KPF_6_ into the 1 M KFSI in DME electrolyte solution decreases the number of solvent molecules surrounding the K ion and simultaneously leads to facile K^+^ de‐solvation. During the electrodeposition process, these unique features can lower the exchange current density between the electrolyte and K‐metal anode, thereby improving the uniformity of K electrodeposition, as well as potentially suppressing dendritic growth. Even under a high current density of 4 mA cm^−2^, the K‐metal anode in 0.05 M KPF_6_‐containing electrolyte ensures high areal capacity and an unprecedented lifespan with stable Coulombic efficiency in both symmetrical half‐cells and full‐cells employing a sulfurized polyacrylonitrile cathode.

## Graphical abstract

The addition of 0.05 M KPF_6_ salt to 1 M KFSI dissolved in a DME electrolyte regulates the solvation sheath structure of K ions. This strategy greatly stabilizes the K‐metal anode by suppressing the dendritic growth of K and minimizing electrolyte depletion in K‐metal batteries, eventually extending the lifetime of K‐metal full batteries.

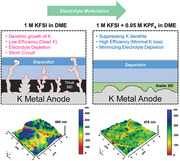

## 1 Introduction

Natural energy resources face radical depletion and environmental pollution is continuously increasing. These matters are the important issues of our time, and the world is at a defining moment.^[^ ^1^, ^2^, ^3^, ^4^, ^5^ ^]^ Therefore, global concerns about energy issues and climate change can no longer be taken lightly. In this regard, there is an ongoing search for sustainable energy substitutes that can guarantee economic growth and further development on a long‐term basis. To date, lithium‐ion batteries (LIB) are the dominant technology and have been located at the center of the endeavor to address the aforementioned issues owing to their high−energy density and satisfactory running time for various electronic applications.^[^ ^6^ ^]^ However, the shortage of lithium and other valuable elements could threaten the energy supply because the most directly available resources are geographically concentrated at limited sites.^[^ ^7^, ^8^ ^]^ Hence, beyond LIBs, other technologies are attracting extensive attention, particularly alkali‐metal batteries, given their high theoretical capacity and the low redox potential of alkali‐metal anodes.^[^ ^9^, ^10^, ^11^, ^12^ ^]^

Rechargeable potassium metal batteries (KMBs) are rapidly gaining scientific attention based on the inherent superiorities of K‐metal anodes, such as low standard electric potential ([Li: −3.04 V vs Na: −2.71 V vs K: −2.93 V] vs SHE) and the high natural abundance of potassium resources (Li: 0.0017 wt.% vs Na: 2.36 wt.% vs K: 2.09 wt.%).^[^ ^13^, ^14^ ^]^ Thus, KMBs have been extensively investigated as a complementary technology to commercial LIBs in the future. Metallic potassium as an anode provides a high specific capacity of 687 mAh g^−1^,^[^ ^15^, ^16^ ^]^ which is almost 2.5 times that of the graphite anode with potassium (279 mAh g^−1^ based on KC_8_),^[^ ^17^ ^]^ and generally surpasses the capacity that can be achieved with most available anodes in rechargeable potassium batteries.^[^ ^13^ ^]^ Owing to the extremely high chemical reactivity of K‐metal, however, its successful utilization in KMBs has been hampered by critical issues. Most importantly, dendrite growth is a ubiquitous key challenge for all alkali–metal anodes, appearing to be especially severe with K‐metal due to its higher reactivity.^[^ ^18^ ^]^ Generally, K dendrites have a porous structure with a mossy or needle‐shaped morphology, which can expose a large surface area of fresh K‐metal, leading to excessive electrochemical reaction with the electrolyte component and a continuous breakdown/re‐construction of the solid electrolyte interphase (SEI) during cycling. Further, fast‐charging rates (high current densities) certainly accelerate K dendrite growth. The resulting K dendrites produce isolated electrochemically inactive “dead K” and can penetrate the separator, eventually leading to earlier cell failure, and can incur further safety hazards.^[^ ^15^, ^19^ ^]^

To address the persistent problems facing K‐metal anodes, some strategies such as electrolyte modulation^[^ ^20^, ^21^, ^22^, ^23^, ^24^, ^25^, ^26^, ^27^, ^28^ ^]^ and the use of a 3D host structure^[^ ^29^, ^30^ ^]^ and/or artificial SEI^[^ ^31^, ^32^ ^]^ are being initially explored. A retrospective look at studies on Li‐metal and/or Na‐metal undoubtedly shows that developing well‐engineered electrolytes with regulated solvation chemistry via the smart combination of salts/solvents/additives is the most effective and critical method of stabilizing K‐metal anodes. This is because the solvation sheath of the potassium ions can be modulated to enable dendrite‐free K deposition and improve the stability of the SEI layer without complicated and time‐consuming procedures. In previous studies on K‐metal anodes, either potassium hexafluorophosphate (KPF_6_) or potassium bis(fluorosulfonyl)imide (KFSI) was used as the main electrolyte salt in carbonate‐ester or ether solvents.^[^ ^1^, ^2^ ^]^ Those studies found that the use of KFSI as a salt is a better choice for cleverly designing an effective electrolyte for reversible K plating/stripping via electrochemistry because the electrolyte can form a robust fluorine‐rich SEI layer and afford dense K deposition on K‐metal surface.^[^ ^25^, ^33^ ^]^ Impressive improvements in the stability of K‐metal anodes using KFSI‐based electrolytes have been achieved; however, the capacity loading and the fast‐charging rates are generally unsatisfactory (≤ 1 mAh cm^−2^ and/or ≤ 1 mA cm^−2^). Even worse, current KMBs have complex battery components and require a large amount of electrolyte solution due to limitations in the current research technology.^[^ ^33^, ^34^, ^35^, ^36^, ^37^, ^38^, ^39^, ^40^, ^41^, ^42^, ^43^, ^44^, ^45^, ^46^, ^47^, ^48^, ^49^ ^]^ Namely, the achievements are still far below those required for high‐energy and high‐power battery applications. In order to further enhance the applicability of KMBs under practical conditions (high capacity loading with fast‐charging rate) and achieve cycling stability of the K‐metal anode, hence, it is no doubt that the development of advanced electrolyte solutions is vital at the current stage.

In this study, we report an important discovery for stabilizing the K‐metal anode by regulating the electrolyte solution. The addition of an optimal amount of 0.05 M KPF_6_ to 1 M KFSI dissolved in 1,2‐dimethoxyethane (DME) improved the uniformity of K electrodeposition and simultaneously enhanced the mechanical rigidity of the SEI layer, thereby significantly suppressing dendritic growth on K‐metal anode surface. The modified electrolyte employing 0.05 M KPF_6_ enables long cycling stability of the K‐metal anode in the K | K symmetrical cells as well as ensures an unprecedented lifespan and stable Coulombic efficiency of a high‐energy K–metal full battery with sulfurized polyacrylonitrile cathode.

## 2 Results and Discussion

### 2.1 Electrolyte Design with Dual‐Salt Combination

**Figure 1:**
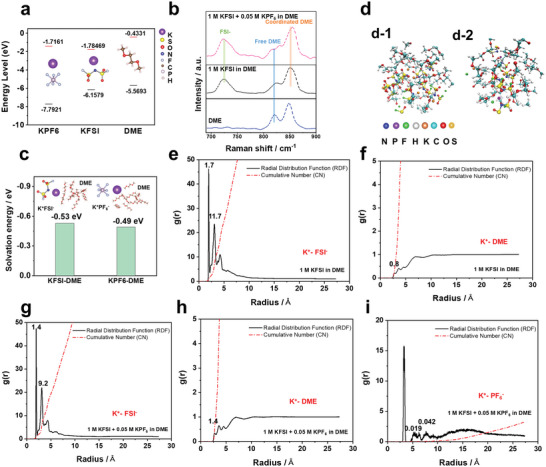
a) The molecular energy levels of the highest occupied molecular orbital (HOMO) and the lowest unoccupied molecular orbital (LUMO) and b) solvation energy of the solvent molecules and potassium salts. c) Raman spectra of DME solvent and electrolyte solutions with/without 0.05 M KPF_6_ in the baseline electrolyte. (1 M KFSI in DME). d) Snapshots 3D MD visualization during MD simulations; d–1) baseline electrolyte (1 M KFSI in DME) and d–2) 0.05 M KPF_6_–containing electrolyte (1 M KFSI + 0.05 M KPF_6_ in DME). RDF and Cumulative numbers of e) K^+^–FSI^−^, f) K^+^–DME in 1 M KFSI in DME, and g) K^+^–FSI^−^, h) K^+^–DME, and i) K^+^–PF_6_^−^ in 1 M KFSI +0.05 M KPF_6_ in DME electrolytes.

**Figure 2:**
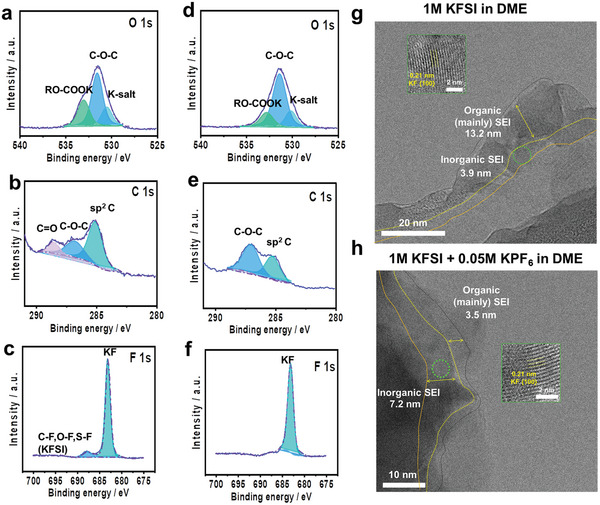
Characterization of the chemical components of SEI layers: a–c) baseline electrolyte and d–f) 0.05 M KPF_6_‐containing electrolyte. XPS spectra of a,d) O 1s, b,c) C 1s, and c,f) F 1s. Low‐temperature (−110 °C) HR‐TEM images of SEI layers on CNT surface: g) baseline electrolyte and h) 0.05 M KPF_6_‐containing electrolyte after 20 cycling at a current density of 1 mA cm^−2^ and capacity loading of 4 mAh cm^−2^. The crystalline KF compound corresponding to the (001) plane was marked as a green square in the inorganic layer.

A solution of 1 M KFSI salt dissolved in DME solvent, which has been widely used as the electrolyte solution in KMBs, was prepared herein as the baseline electrolyte.^[^ ^44^, ^45^, ^46^, ^47^ ^]^ In this study, we designed an advanced electrolyte by modulating the baseline electrolyte with KPF_6_ salt. In order to confirm the fundamental properties of the modified electrolyte, density functional theory (DFT) calculations and Raman spectroscopy analysis were initially conducted. First, the molecular energy levels such as lowest unoccupied molecular orbital (LUMO) and highest occupied molecular orbital (HOMO) energy levels of the solvent and salts for the modified electrolyte are displayed in **Figure** **1**a, respectively. KFSI salt has a lower LUMO energy level than that of KPF_6_ and DME, suggesting that KFSI salt readily decomposed during the electrochemical reduction (plating) process and plays a key role in determining the chemical components of the SEI layer. Corresponding to the lower HOMO level of KPF_6_ than KFSI salt, we also confirmed that the addition of KPF_6_ in the electrolyte can improve the high voltage stability of the baseline electrolyte from linear sweep voltammetry analysis (Figure S1, Supporting Information). The solvation energies between KPF_6_ and DME and KFSI and DME were calculated, respectively, and compared in Figure 1b. It is confirmed that KPF_6_‐DME (−0.49 eV) has a lower solvation than that of KFSI–DME (−0.53 eV); this implies that the addition of 0.05 M KPF_6_ in the baseline electrolyte can facilitate the K^+^ desolvation and diffusion kinetics during the plating process.^[^ ^50^, ^51^ ^]^ Raman spectroscopy analysis showed that the incorporation of 0.05 M KPF_6_ into the baseline electrolyte changed the K^+^ solvation structure (Figure 1c). In the Raman spectrum of DME solvent, two peaks were observed at 820 and 848 cm^−1^, which can be assigned to the rocking vibration of CH_2_ and the stretching vibration of C–O, respectively. After adding 0.05 M KPF_6_, the proportion of solvated DME in the baseline electrolyte increased. In the modified electrolyte, the proportion and intensity of the peak at 854 cm^−1^ corresponding to coordinated DME (K^+^‐solvated DME) were higher than that for the baseline electrolyte.^[^ ^35^ ^]^ This demonstrates that the addition of 0.05 M KPF_6_ lowers the proportion of free solvent in the baseline electrolyte. In addition, the statistical number of the coordinated solvents for baseline and modified electrolytes were precisely analyzed using molecular dynamics (MD) simulation.^[^ ^52^, ^53^ ^]^ We first optimized the molecular structure (Figure 1d–1,2) and then calculated the coordination number of K^+^ with FSI^−^ and DME either in 1 M KFSI in DME electrolyte and 1 M KFSI in DME with the addition of 0.05 M KPF_6_. Figure 1e–i presented the g(r) of the value corresponding to the radius of K^+^ with FSI^−^, K^+^ with PF_6_^−^, and K^+^ with DME through RDF (black line). The g(r) is the probability of finding a particle at a distance r from another tagged particle. Therefore, we can know how much DME solvent is combined around K^+^ through the cumulative number (red line) corresponding to each radius value, which has a similar meaning to the coordination number. For 1 M KFSI in DME electrolyte (Figure 1e), the coordination numbers of K^+^ with FSI^−^ at first solvation shell (0–2.16 Å) are 1.7 and 11.7 at second solvation shell (2.16–3.6 Å), meanwhile, the coordination number of K^+^ with FSI^−^ at first solvation shell are 1.4 and 9.2 at second solvation shell in 1 M KFSI in DME with the addition of 0.05 M KPF_6_ (Figure 1g). The coordination numbers of K^+^ with PF_6_^−^ at the first solvation shell (0–3.64 Å) are 0.019 and 0.042 at the second solvation shell (4.64–6.76 Å) (Figure 1i). For coordination number of K^+^ with DME at 1 M KFSI in DME only have 0.8 (Figure 1f), otherwise the coordination number of K^+^ with DME at 1 M KFSI in DME with the addition of 0.05 M KPF_6_ has 1.4 coordinated number (Figure 1h). A combination study of Raman spectroscopy, DFT, and MD simulations suggests that the addition of 0.05 M KPF_6_ in 1 M KFSI in DME electrolyte increased the coordination number for K^+^ with DME which could facilitate desolvation.

### 2.2 Formation of Stable SEI Layer on K‐Metal Anode

The formation of a stable SEI layer is one of the key factors for stabilizing the K‐metal anode because a robust SEI can effectively suppress parasitic reactions between the electrolyte and K‐metal anode, including dendritic growth of K and electrolyte consumption. To investigate the composition of the SEI layers formed with different electrolytes (with/without 0.05 M KPF_6_), initially, X‐ray photoelectron spectroscopy (XPS) analysis was carried out of the K‐metal anode after 5 cycles in asymmetric K | Cu cells (**Figure** **2**). For both electrolytes, inorganic/organic complexes such as KF, =C–O–C, and C=O were observed in the SEI layer; these compounds are generally formed by the decomposition of the KFSI salt and DME solvent.^[^ ^35^ ^]^ The O 1s spectrum was deconvoluted into three peaks, where the two peaks at 532.9 eV (RO–COOK) and 531.5 eV (C–O–C) are associated with the decomposition of DME solvent and that at 530 eV is derived from the reduction of the KFSI salt (Figure 2a and d).^[^ ^54^ ^]^

The C 1s spectrum was deconvoluted into three peaks with binding energies of 284.8 (C–C), 286.4 (C–O–C), and/or 287.8 eV (C=O), which are attributed to the reduction of the solvent (Figure 2b,e).^[^ ^55^ ^]^ In the case of the 0.05 M KPF_6_–containing electrolyte, notably, the relative intensity of the peak related to RO–COOK was reduced and no C=O peak was observed in the O 1s and C 1s spectra, respectively, indicating less solvent decomposition. This is because the addition of 0.05 M KPF_6_ can increase the number of coordinated solvent molecules around the K ions in the baseline electrolyte. The N 1s spectra (Figure S2, Supporting Information) further demonstrate a salt reduction in the baseline electrolyte. The P 2p spectrum of the 0.05 M KPF_6_–containing electrolyte revealed the reduction of the KPF_6_ salt (Figure S3, Supporting Information). The strong peak at 683.3 eV in the F 1s spectrum, corresponding to KF, was the primary peak for both samples, and it is attributed to the reduction of KFSI salt (Figure 2c,f).^[^ ^37^ ^]^ However, interestingly, a relatively large fraction of KF in the 0.05 M KPF_6_‐containing electrolyte was formed by additional reduction of the PF_6_^−^ (see the P 2p spectrum in Figure S3, Supporting Information); this can be supported by facile desolvation kinetics of K^+^–PF_6_^−^ in DME during electrodeposition reaction (Figure 1b).^[^ ^56^ ^]^ On the other hand, an additional peak at 687.4 eV, corresponding to C–F, O–F, and S–F (KFSI), was also observed in the F 1s spectrum of the baseline electrolyte due to some extent of salt/solvent decomposition. The addition of 0.05 M KPF_6_ to the baseline electrolyte enhanced the formation of a KF‐rich SEI layer, which provides mechanical strength and thus helps to suppress the dendritic growth of K and large volume changes of the K‐metal anode during multiple plating/stripping processes. The XPS results for the SEI layers formed with different electrolytes were corroborated by Raman spectroscopy. To further verify the thickness and organic/inorganic components of the SEI layer, therefore, HR‐TEM (high‐resolution transmission electron microscopy) analysis of the SEI layer was conducted at a low temperature of −110 °C. For HR‐TEM analysis, all samples were carefully prepared using an asymmetric cell comprising of K‐metal as anode and free‐standing carbon‐nanotubes film as K^+^ host (K/CNT).^[^ ^57^ ^]^ In K/CNT cells, K‐metal was deposited and stripped on CNT film over 20 cycles at a current density of 1 mA cm^−2^ and capacity loading of 4 mAh cm^−2^ (Figure S4, Supporting Information). Then, after stripping at the 20^th^ cycle, the collected SEI on CNT film was used for HR‐TEM analysis. As seen in Figure 2g,h, the baseline electrolyte produced a thicker SEI than the modified electrolyte. For both electrolytes, the crystalline KF compound corresponding to the (001) plane was indexed.^[^ ^57^ ^]^ In addition to XPS data, it was clearly proved that SEI induced by modified electrolyte was composed of a thicker inorganic layer with a large fraction of crystalline grains than SEI induced by baseline electrolyte. In addition, we carried out fast Fourier transform (FFT) analysis for HR‐TEM data for the collected SEI on the CNT film and the results are displayed in Figure S5, Supporting Information. It should be noted that we could not collect the clear crystal phase of K‐metal from the FFT image because the metallic potassium is covered by the SEI layer and CNT film. Nonetheless, the FFT image in Figure S5b, Supporting Information from the red square region in Figure S5a, Supporting Information showed a series of concentric rings of the BCC crystal structure of potassium. Furthermore, the FFT data collected from two regions (black and blue square) in Figure S6, Supporting Information showed markedly different diffraction patterns. It also could be noted that we can't collect the clear diffraction patterns of organic and inorganic SEI compounds because the SEI layer on CNT film consisted the various complexes induced by electrolyte decomposition including K‐metal. FFT image in Figure S6b, Supporting Information (collected from black square) revealed randomly scattered patterns corresponding to the organic compounds. In contrast, the FFT image in Figure S6c, Supporting Information (collected from blue square) revealed regularly spaced lattice patterns or grid patterns corresponding to inorganic compounds. Overall, the results suggest that the addition of 0.05 M KPF_6_ in the baseline electrolyte can mitigate solvent and/or salt decomposition and further enhance the stability of the SEI layer on the K‐metal surface.

### 2.3 Structural Evolution of Electrodeposited K‐metal

**Figure 3:**
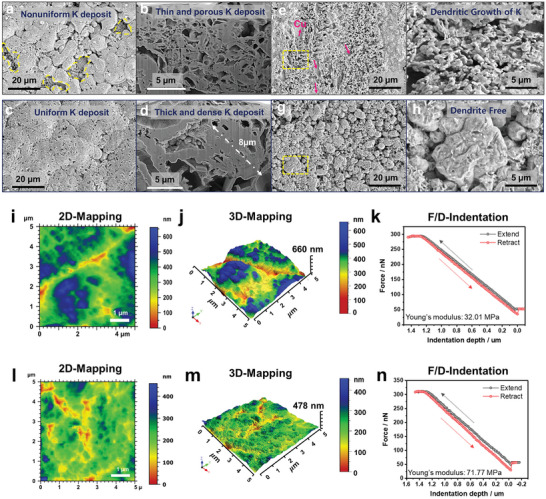
Top‐view and cross‐sectional SEM images of deposit K‐metal cycled at a current density of a,d) 1 and e—h) 4 mA cm^−2^ with capacity loading of 1 mAh cm^−2^: a,b,e,f) baseline electrolyte and c,d,g,h) 0.05 M KPF_6_‐containing electrolyte. 2D and 3D AFM topographic images (5 × 5 µm^2^ scan size) of electrodeposited K on the Cu substrate at a current density of 4 mA cm^−2^ and capacity loading of 1 mAh cm^−2^ in i,j) baseline electrolyte and l,m) 0.05 M KPF_6_‐containing electrolyte. Force‐displacement curve of SEI layer on Cu substrate after stripping at 4 mA cm^−2^ and 1 mAh cm^−2^ in k) baseline electrolyte and n) 0.05 M KPF_6_‐containing electrolyte.

Structural evolution of the electrodeposited K was observed using scanning electron microscope (SEM) analysis with a focused ion beam (FIB), where K | Cu asymmetric cells employing the baseline and 0.05 M KPF_6_‐containing electrolytes were assembled. The deposition of K–metal on the Cu–foil at current densities of 1.0 and 4 mA cm^−2^ was investigated after the disassembly of the cells. The SEM images in **Figure** **3**a–d showed that at 1.0 mA cm^−2^, the 0.05 M KPF_6_‐containing electrolyte produced a uniform K‐deposit film compared to the baseline electrolyte. Cross‐sectional samples of the deposited K were prepared using FIB. The cross‐sectional SEM images were precisely compared, showing that the 0.05 M KPF_6_‐containing electrolyte (Figure 3c,d) led to a dense potassium deposit with a morphology comprising almost fully connected thick particles (particle size: around 8 µm), whereas the deposited potassium metal cycled in the baseline electrolyte formed relatively thin discrete particles with a porous structure (Figure 3a,b). This trend became more pronounced at the higher current density of 4.0 mA cm^−2^. Although the K‐deposit film had a relatively loose morphology than that of the sample obtained at low current density (1 mA cm^−2^), potassium was uniformly deposited on the entire Cu‐foil in the 0.05 M KPF_6_‐containing electrolyte (Figure 3g,h). In contrast, due to the poor kinetics of potassium plating, the baseline electrolyte produced an unevenly distributed electrical field at high current density, resulting in dendritic potassium fibers (Figure 3e,f). As a result, the potassium film deposited from the baseline electrolyte cell was irregular and porous with nanosized dendritic potassium.

In addition to SEM measurement data, we further investigated the differences in potassium plating morphologies and mechanical strength of SEI layers on Cu‐foil depending on with/without 0.05 M KPF_6_ in the baseline electrolyte through atomic force microscopy (AFM) analysis. AFM is advantageous in this aspect because of its high‐resolution imaging as well as force probing capabilities along the surface normal on metallic anode. First, topographic AFM images and corresponding thickness histogram of deposited K in baseline and 0.05 M KPF_6_‐containing electrolyte were compared in Figure 3i–n and Figure S7, Supporting Information, which demonstrated the drastic differences in height variation and roughness. The 0.05 M KPF_6_‐containing electrolyte produced the uniform K–deposit morphologies with an average thickness of ≈300–400 nm (Figure 3l,m) whereas the baseline electrolyte showed the non–uniform K–deposit morphologies with large fluctuation in average thickness between ≈100–700 nm (Figure 3i,j). Moreover, we evaluated the mechanical strength of the SEI layer through the AFM force displacement (F/D) measurements. Both electrolytes showed typical F/D curves of the SEI layer (Figure 3k,n); however, noticeable differences in the mechanical strength of the SEI layer are observed from Young's modulus.

The Hertz model that is applicable in the case of small tips and stiff samples with a small adhesion is preferably adopted for calculating Young's modulus of the SEI according to the following equation.

(1) $$F\;=\frac{4}{3}\;\frac{E}{\left(1-\theta^{2}\right)}\sqrt{R}{\left(d_{max}-d\right)}^{\frac{3}{2}}-F_{max}$$

where *E*, *θ*, *d*, *F*, and *R* are the Young's modulus of the surface, the Poisson's factor of the surface (0.5), adhesion distance, adhesion force, and the curvature radius of the probe (≈10 nm), respectively, and the *d_max_* and *F_max_* are the point of maximal adhesion distance and the adhesion force for retract curve.^[^ ^58^, ^59^, ^60^ ^]^ The SEI layer with 0.05 M KPF_6_‐containing electrolyte showed a higher Young's modulus (7.613 GPa) than with baseline electrolyte (6.708 GPa). This clearly indicated that the SEI layer induced by the 0.05 M KPF_6_‐containing electrolyte contained a relatively larger fraction of KF compound compared to the baseline electrolyte. Such AFM results are in good agreement with the combination study of DFT, MD, Raman, XPS, and TEM analysis that the addition of 0.05 M KPF_6_ in the baseline electrolyte provides effective functionalities to suppress the dendritic growth of K.^[^ ^61^, ^62^, ^63^, ^64^ ^]^

**Figure 4:**
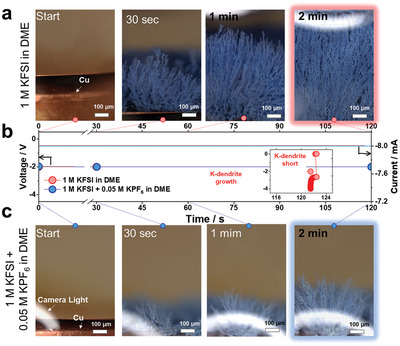
Snapshots from operando optical microscopy observation of electrochemical K deposition on Cu substrate as a function of time: a) baseline electrolyte and c) 0.05 M KPF_6_‐containing electrolyte. b) Voltage versus time profiles during the electrodeposition process at a current density of 8 mA cm^−2^ and capacity loading of 8 mAh cm^−2^.

Operando optical microscopy (OM) analysis provided solid evidence that the addition of 0.05 M KPF_6_ to the baseline electrolyte effectively suppressed the growth of potassium dendrites. Operando OM observations were performed under dynamic conditions using the customized electrochemical cell (Figure S8, Supporting Information). The growth of potassium dendrites was observed within a short period of time at a current density of 8 mA cm^−2^ and capacity loading of 8 mAh cm^−2^ during the plating process. As seen in the supporting movie and snapshots from the movie (Movie S1, Supporting Information: baseline electrolyte, Movie S2, Supporting Information: 0.05 M KPF_6_–containing electrolyte and **Figure** **4**a–c), K dendrites grew much faster in the baseline electrolyte than in the 0.05 M KPF_6_–containing electrolyte during the initial 30 s. After 2 min, the K dendrites in the baseline electrolyte eventually reached the counter‐electrode, leading to an electrical short‐circuit, whereas the 0.05 M KPF_6_‐containing electrolyte significantly delayed the growth of dendritic K.

Exchange current density is a key parameter for describing the intrinsic kinetics of electron transfer at the electrode in the reduction of K^+^ to K, and thus, critically affects the electrodeposition of potassium metal with different electrolytes.^[^ ^65^ ^]^ As shown in Figure S9, Supporting Information, the exchange current density of the potassium electrode in the electrolyte comprising 1 M KFSI + 0.05 M KPF_6_ in DME is 0.77 µA cm^−2^, and that employing the electrolyte comprising 1 M KFSI in DME is 1.14 µA cm^−2^. Based on these experimental results, phase‐field modeling coupled with cellular automaton was used to probe the electrodeposition of potassium metal with different electrolytes. The reaction describing the deposition of potassium metal can be expressed as.

(2) $$K^{+}+e^{-}↔K$$

Calculations for the above electrochemical reaction were performed by applying the Butler–Volmer equation to describe the relationship between the overpotential and local current density, which are bridged by the exchange current density:

(3) $$J\;=\vec{j}\;-\;\overset{\leftarrow }{j}=j_{0}\;\left[\exp\left(\frac{\alpha F}{\mathrm{RT}}\eta_{c}\right)-\exp\left(-\frac{\beta F}{\mathrm{RT}}\eta_{c}\right)\right]$$

where *J*, *j*_0_, and *η* are the Faradic current density, exchange current density, and overpotential, respectively. *F* is the Faradic constant of 96 485 C mol^−1^. R is the molar gas constant of 8.314 J mol^−1^ K^−1^, and *T* is the temperature of 25 °C. Moreover, *α* and *β* are the anodic and cathodic charge‐transfer coefficients, where the condition *α* + *β* = 1 holds for a single‐electron transfer reaction.

**Figure 5:**
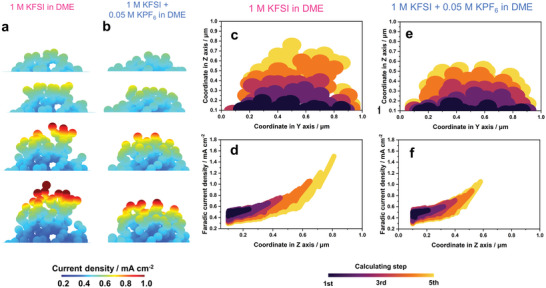
Morphological evolution of K‐metal in an electrolyte comprising a) baseline electrolyte and b) 0.05 M KPF_6_‐containing electrolyte. The color on the structure represents the local Faradic current density. Cross‐section of simulated morphology of K‐metal electrode showing evolution in an electrolyte comprising c) baseline and e) 0.05 M KPF_6_‐containing electrolytes. Distribution of Faradic current density on K‐metal with electrolyte comprising d) baseline and f) 0.05 M KPF_6_‐containing electrolytes.

The morphological evolution of potassium metal during the electrodeposition process with different electrolytes is shown in **Figure** **5**, and Figures S10 and S11, Supporting Information. Potassium metal was uniformly electrodeposited in the electrolyte comprising 1 M KFSI + 0.05 M KPF_6_ in DME, with a dense morphology having no obvious internal holes (Figure 5b,e and Figure S10b, Supporting Information). The distribution of current density on the entire layer of deposited potassium was relatively uniform, further indicating even electrodeposition in the following step. Nevertheless, electrodeposition of potassium with the baseline electrolyte led to a non–uniform distribution of the local current density and formed loose dendrites with highly branched structures (Figure 5a,c and Figure S10a, Supporting Information).

These features show in good agreement with AFM topographic data in Figure 3j,m. The distribution of Faradic current density with different electrolytes is shown in Figure 5d,f, where the concentration of the local current density will induce the electrodeposition of dendritic potassium. The current distribution profile of electrodeposited K was quantitatively derived to analyse the morphological evolution. As shown in Figure 5c,e and Figure S10, Supporting Information, electrodeposition of K with the addition of 0.05 M KPF_6_ led to a more regular quasi‐rectangular shape with a lower aspect ratio. Therefore, the electrolyte comprising 1 M KFSI + 0.05 M KPF_6_ in DME with a lower exchange current density improves the uniformity of K electrodeposition and can potentially suppress dendritic growth, which is expected to prolong the cycle‐life of the K‐metal anode.

### 2.4 Enhanced Electrochemical Properties of K‐Metal Anode

The introduction of 0.05 M KPF_6_ in the baseline electrolyte is a very simple strategy but has great potential for enhancing the stability of potassium metal. The previously reported KMBs were generally fabricated under loose experimental control; in particular, using two or more separators (i.e., a combination of polymer and glass fiber separators), and a large fraction of electrolyte (flood condition) was employed because of the serious uncontrollable dendrite problems (Table S1, Supporting Information). To clearly confirm the energetic effect of electrolyte modulation on the stability of the K‐metal electrode, herein, a single polymer separator (Celgard 2400) was utilized, with a relatively small amount of electrolyte (100 or 50 µL). To determine the K^+^ conductivity in the electrolyte solution, the electrochemical impedance spectroscopy (EIS) measurement (Figure S12, Supporting Information) and the following equations are used:^[^ ^66^ ^]^ the 0.05 M KPF_6_‐containing electrolyte exhibited the higher K^+^ conductivity (2.321 × 10^−3^ S cm^−1^) compared to the baseline electrolyte (1.717 × 10^−3^ S cm^−1^); this could lead to uniform K^+^ flux distribution on K‐metal surface, consequentially suppressing the K dendrite growth. The importance of the solvation structure was confirmed by studying K plating/stripping in asymmetric K | Cu cells. Potassium metal with a capacity of 2 mAh cm^−2^ was plated on Cu‐foil at 2 mA cm^−2^ and the plated K was stripped at 1.0 V during 50 cycles (Figure S13a, Supporting Information). The voltage profiles show significant differences with increasing cycle numbers. During 20 cycles, the baseline electrolyte showed very unstable plating/stripping behavior, with significant noise and large voltage polarization (Δ*V*= 1.064 V) in the voltage profile. This is because once the dendrite is formed during the plating process, the SEI layer should be broken and this re‐exposes the fresh K‐metal surface to the electrolyte, and the SEI forms again, accelerating electrolyte depletion. Finally, these side reaction chains result in increased thickness of the SEI upon repeated plating/stripping and thus slows K ion diffusion. In contrast, the modified electrolyte with 0.05 M KPF_6_ exhibited very stable plating/stripping reactions with small voltage polarization (Δ*V*= 0.287 V) during 50 cycles (Figure S13b, Supporting Information). The differences in the K plating/stripping reaction in K | Cu cells directly mirrored the EIS data. The obtained AC impedance spectra consist of impedance from the electrolyte, the electrode SEI layer at the K‐metal surface, and the charge‐transfer reaction. As expected from the different voltage profiles in the K | Cu cells, the 0.05 M KPF_6_‐containing electrolyte exhibited much lower resistance than the baseline electrolyte, indicating the formation of a stable SEI layer and facile K ion diffusion kinetics (Figure S13c, Supporting Information). The average Coulombic efficiency (CE) of the potassium electrode was determined using the modified method reported by Zhang et al., in order to determine the amount of potassium lost during cycling.^[^ ^67^ ^]^ This method is designed to minimize the effect of the Cu substrate in K | Cu cells. The constant‐current protocol and voltage‐time plot used for calculating the CE values are included in Figure S14, Supporting Information. The average CE values over n cycles were calculated using the following equation:

(4) $$CE_{avg}=\frac{nQ_{C}+Q_{S}}{nQ_{C}+Q_{T}}$$

where the number of cycles (*n*) is 50. The average CEs for the baseline electrolyte and 0.05 M KPF_6_‐containing electrolyte were 97.2% and 99.3%, respectively. This difference indicates that the addition of 0.05 M KPF_6_ to the baseline electrolyte improves the K plating/stripping reaction kinetics with minimal loss of potassium during cycling. To understand the effect of 0.05 M KPF_6_ in the modified electrolyte on the cycling behavior of the K | Cu asymmetric cell, the cycled K‐metal anodes and separator collected from the asymmetric cells after 20 cycles (for the baseline electrolyte) and 50 cycles (for the 0.05 M KPF_6_‐containing electrolyte) were investigated. As seen in the top‐view and cross‐sectional SEM images (Figure S15, Supporting Information), the K‐metal anode cycled in the baseline electrolyte had a very loose structure with potassium dendrites, and corrosion of the fresh K‐metal was observed. In contrast, the K‐metal anode cycled in the 0.05 M KPF_6_‐containing electrolyte had a dendrite‐free morphology and dense structure. In addition, the 0.05 M KPF_6_‐containing electrolyte showed a smooth surface without the formation of dead K after the stripping reaction (Figure S16, Supporting Information). Moreover, some areas of the separator cycled in the baseline electrolyte had melted and blocked the pores (Figure S17, Supporting Information). This is because internal short‐circuiting continuously progressed due to the dendritic growth of K upon cycling, resulting in serious degradation of the separator surface. In contrast, the separator cycled in 0.05 M KPF_6_‐containing electrolyte retained the original morphology and there was no severe damage.

**Figure 6:**
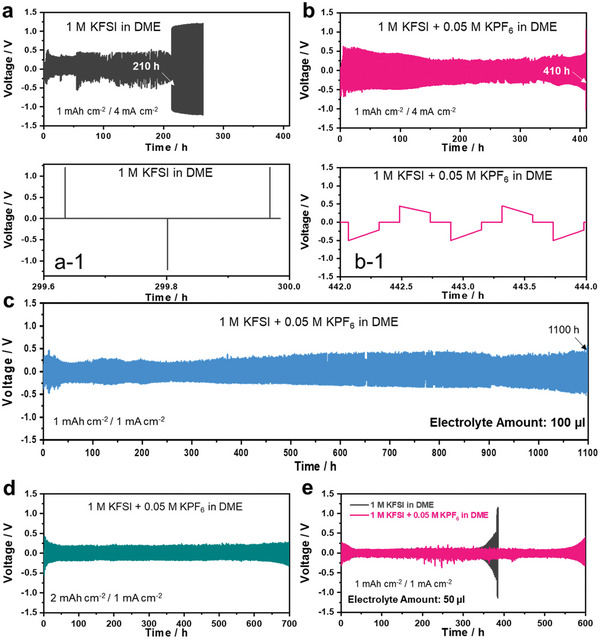
Galvanostatic cycling test of K | K symmetric cells with/without 0.05 M KPF_6_ in the baseline electrolyte at a current density of 4 mA cm^−2^ and capacity loading of 1 mAh cm^−2^ using 100 µL of electrolyte: a and a‐1) baseline electrolyte and b and b‐1) with 0.05 M KPF_6_‐containing electrolyte_._ c) Galvanostatic cycling test of K | K symmetric cells with 0.05 M KPF_6_‐containing electrolyte at 1 mA cm^−2^ and 1 mAh cm^−2^ using 100 µL of electrolyte. d) 0.05 M KPF_6_‐containing electrolyte cycled at 1 mA cm^−2^ and 2 mAh cm^−2^ using 100 µL of electrolyte. e) Galvanostatic cycling test of K | K symmetric cells with/without 0.05 M KPF_6_ in the baseline electrolyte at 1 mA cm^−2^ and 1 mAh cm^−2^ with 50 µL of electrolyte. All cells are used a single separator without a glass fiber filter.

To realize the practical application of KMBs, the electrolyte solution must exhibit electrochemical stability and good compatibility with the K–metal anode. Along with asymmetric K | Cu cells, galvanostatic cycling was performed using symmetric K | K cells at various current densities and cycling capacities. All cells were fabricated with one polymer separator, Celgard 2400, and a limited amount of electrolyte (100 or 50 µL). The K | K symmetric cell was initially tested under a very high current density of 4 mA cm^−2^ and capacity loading of 1 mAh cm^−2^ (corresponding to 0.5 h per cycle). The K | K symmetric cell employing the baseline electrolyte showed unstable plating/stripping behavior, eventually failing after 210 h (**Figure** **6**a and a‐1 In contrast, with the 0.05 M KPF_6_‐containing electrolyte, the cell demonstrated excellent cycling stability for 410 h (Figure 6b and b‐1). Moreover, the K | K cells with 0.05 M KPF_6_‐containing electrolyte demonstrated unprecedented cycling stability under various constraints. At a current density of 1.0 mA cm^−2^ and capacity loading of 1.0 mAh cm^−2^, the K | K cell exhibited unprecedented long–term cycling stability and a low overpotential over 1100 h (Figure 6c). The improvement in the cycling stability upon using the 0.05 M KPF_6_‐containing electrolyte was further verified under practical test protocols. With a smaller amount of electrolyte (50 µL), the lifetime of the K | K symmetric cells employing the 0.05 M KPF_6_‐containing electrolyte was extended to over 600 h (Figure 6e). Even under a high capacity loading of 2.0 mAh cm^−2^ (current density of 1.0 mA cm^−2^), the K | K cell maintained a stable polarization voltage and could be cycled over 700 h in the 0.05 M KPF_6_‐containing electrolyte (Figure 6d). On the other hand, the optimal composition of KPF_6_ salt to modulate the baseline electrolyte was further investigated through the galvanostatic cycling test of the K | K symmetric cell upon using 0.1 M KPF_6_‐containing electrolyte (Figure S18, Supporting Information). Although the K | K symmetric cell can survive over 350 h with 0.1 M KPF_6_‐containing electrolyte at a current density of 1 mA cm^−2^ and capacity loading of 2 mAh cm^−2^, the lifetime of the K | K symmetric cell was only half that of using the electrolyte containing 0.05 M KPF_6_. The reason is that as the mole fraction of KPF_6_ salt in the baseline electrolyte increases, the decomposition level of the PF_6_^−^ and DME solvent can be also increased during the plating process because of the relatively weak solvation energy of K^+^PF_6_^−^‐DME than that of K^+^FSI^−^‐DME complex (see the DFT calculations in Figure 1a). These results are evidenced by the XPS analysis data of the SEI layer on the K‐metal anode induced by the 0.1 M KPF_6_‐containing electrolyte (Figure S19, Supporting Information).

**Figure 7:**
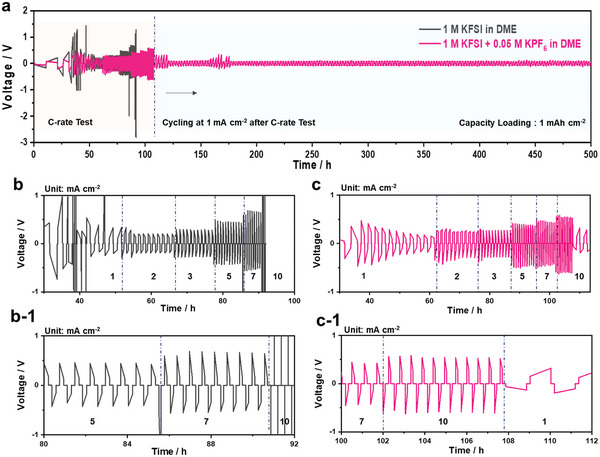
a) C‐rate test with galvanostatic cycling test for K | K symmetric cells with/without 0.05 M KPF_6_ in the baseline electrolyte at a capacity loading of 1 mAh cm^−2^: b and b‐1) baseline electrolyte and c and c‐1) 0.05 M KPF_6_–containing electrolyte. All cells are used a single separator without a glass fiber filter.

Compared to the chemical components in the SEI layer for 0.05 M KPF_6_‐containing electrolyte, the relatively larger fraction of organic compounds is observed from the C 1s and O 1s spectra while the P–F bonds are only detected in P 2p spectra due to the higher decomposition level of DME and PF_6_^−^. Noted that even worse, the presence of P–F bonds (reduction products of PF_6_^−^) can easily produce the corrosive HF through the hydrolysis reaction with a trace amount of water in the electrolyte solution, further accelerating the cell failure.^[^ ^68^, ^69^ ^]^ Finally, the effect of the addition of 0.05 M KPF_6_ to the baseline electrolyte on the rate capability of the K | K cell cells was evaluated (**Figure** **7**). The 0.05 M KPF_6_‐containing electrolyte greatly enhanced the K plating/stripping kinetics up to a high current density of 10 mA cm^−2^ (corresponding to 12 min per cycle), and thereafter, the cell operated continuously over 500 h when the current density was restored to 1 mA cm^−2^.

### 2.5 High‐Performance K‐Metal Batteries

**Figure 8:**
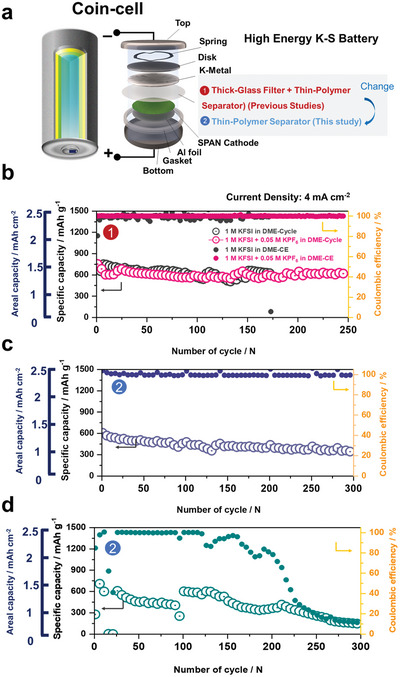
a) Schematic illustrations of K–S battery design used in this work. Comparison of cycling stability of K–S batteries with/without glass filter at a high current density of 4 mA cm^−2^: b) both baseline and 0.05 M KPF_6_‐containing electrolytes with a glass filter and c) 0.05 M KPF_6_‐containing electrolyte without glass filter and d) baseline electrolyte without glass filter.

The ultimate goal in the development of a stable K‐metal anode is its practical use in rechargeable potassium batteries. Recently, potassium‐sulfur (K–S) batteries have attracted substantial interest as one of the KMBs because of the abundance of potassium and low associated costs. Herein, K–S batteries were fabricated using a pyrolyzed polyacrylonitrile‐sulfur (SPAN) cathode. SEM imaging revealed that the SPAN particles have a spherical morphology with an average diameter of 100–150 nm (Figure S20a, Supporting Information). From the SEM energy dispersive X‐ray elemental mapping data, the sulfur content of SPAN was determined to be 45.5 wt.%, and the C/N weight ratio was 2.56, which is close to the theoretical value for six‐membered‐ring‐based poly‐annealed pyridine (C/N = 2.6) (Figure S20b–d, Supporting Information).^[^ ^70^ ^]^ The Fourier transform infrared (FTIR) spectroscopy and Raman spectroscopy data showed the typical SPAN structure comprising a covalently fixed sulfur in the PAN structure (Figure S21). Owing to the covalent and confined S in the SPAN structure, neither polysulfide dissolution nor the detrimental shuttle effect on the metal side was observed; hence, it is undisputed that this material is a good candidate for practical K–S batteries. To confirm the effectiveness of the modified electrolyte in a K–S battery, K–S batteries with/without a glass filter (**Figure** **8**a) were fabricated and tested. First, the K–S battery was assembled with both Celgard 2400 and a glass filter as a separator. In the case of the battery employing the 0.05 M KPF_6_‐containing electrolyte, a high capacity of 800 mAh g^−1^ was obtained, where the CE and capacity retention were as high as 99.9% and 90% over 300 cycles even at a high current rate of 4 mA cm^−2^, respectively. With the baseline electrolyte, the K–S battery also delivered a similar capacity and cycling behavior; however, severe fluctuation in the CE was observed after the initial few cycles (Figure 8b). Without the glass filter, notably, the K–S battery delivered a high discharge capacity and a good lifetime of over 300 cycles with the 0.05 M KPF_6_‐containing electrolyte, whereas the battery with the baseline electrolyte experienced abnormal cycling behavior during the initial few cycles, followed by capacity failure with a very low CE after 150 cycles (Figure 8c,d). To determine the main reason for the difference in the cycling stability depending on the electrolyte solution, post–mortem analysis of the cycled SPAN cathodes was conducted. From Raman spectroscopy, it was observed that the original structure of the cycled SPAN cathode was well retained in both electrolytes (Figure S22, Supporting Information). In addition, the re‐cycled cell employing the cycled SPAN cathode with fresh electrolytes and K‐metal anode exhibited marginal differences in the reversible capacity and cycling stability (Figure S23, Supporting Information). The results clearly demonstrate that the difference in the cycling stability is mainly attributed to the unstable interface of the K‐metal anode induced by the dendritic growth of K on the anode surface. In brief, the good capacity and cycling stability of the K–S battery clearly suggest the commercial viability of the proposed electrolyte, 1 M KFSI + 0.05 M KPF_6_ in DME for practical battery applications.

## 3 Conclusion

A simple and effective electrolyte modulation strategy was employed to stabilize the K‐metal anode. 1 M KFSI dissolved in DME, which is widely used as an electrolyte in KMBs (baseline electrolyte), was modified by adding the 0.05 M KPF_6_. The modified electrolyte has a lower level of free solvent molecules and leads to facile K^+^ desolvation and enables dense and uniform potassium deposition during the plating process. Moreover, the addition of 0.05 M KPF_6_ to the baseline electrolyte mitigates the decomposition of the solvent molecules and leads to form a larger fraction of the KF compound, which enhances the mechanical rigidity of the SEI layer on the surface of the K‐metal electrode. Compared to prior studies reported so far, our work highlights the ultra‐high cycling stability of the K‐metal anode (Table S1, Supporting Information). Moreover, the proposed electrolyte demonstrates excellent practical applicability in a K–S full battery, affording long‐term cycling stability with high CE over 300 cycles. Although K‐metal is still difficult to handle and directly use in practice as an anode due to high sensitivity against air and moisture, we believe that the present strategy, combined with the easy and low‐cost process, should advance the development of high‐energy and safe K‐metal battery applications.

## 4 Experimental Section

### Electrolyte Preparation

Potassium bis(fluorosulfonyl)imide (KFSI, TCI) and potassium hexafluorophosphate (KPF_6_, TCI) were respectively dried under vacuum at 80 and 110 °C using a vacuum oven (Buchi). 1,2–Dimethoxyethane (DME, TCI) was purified using vacuum–dried 4 Å molecular sieves over the course of 3 days. The baseline electrolyte was prepared by dissolving 1 M KFSI in DME. The baseline electrolyte was modified by adding 0.05 _6_ and 0.1 M KPF_6_. In this work, the unit of mol kg^−1^ was denoted as M.

### Cathode Preparation for Full–Cell Test

Polyacrylonitrile (PAN) (Sigma–Aldrich) was homogeneously mixed with elemental sulfur (PAN:sulfur = 1:4 weight ratio). The PAN and sulfur mixture was converted to sulfurized polyacrylonitrile (SPAN) powder by calcination at 350 °C for 6 h under argon gas.

### Electrochemical Measurements

For the K | K symmetric–cell tests with a variation of the areal capacity, current density, and amount of electrolyte, the cell was assembled with 15–*π* potassium metal foil and conducted using the VMP3 (Bio‐Logic) instrument set for galvanostatic cycling (current density: 1 and 4 mA cm^−2^, areal capacity: 1 and 4 mAh cm^−2^, amount of electrolyte: 50 and 100 µL per cell). The rate performance also was investigated using the K | K symmetric‐cell assembly method mentioned above at 1, 2, 3, 5, 7, and 10 mA cm^−2^ and 1 mAh cm^−2^. To observe HR‐TEM images of thickness of SEI layers formed by baseline electrolyte and 0.05 M KPF_6_‐containing electrolyte, K | CNT asymmetric cells were assembled with 15–*π* potassium metal foil and free‐standing CNT film at a current density of 1 mA cm^−2^ and an areal capacity of 4 mAh cm^−2^ for 20 cycles. K | Cu asymmetric cells were fabricated with 15–*π* potassium metal foil and Cu–foil using VMP3 (Bio‐Logic) at a current density of 1 mA cm^−2^ and areal capacity of 1 mAh cm^−2^. Electrochemical impedance spectroscopy (EIS) measurements were performed over the frequency range of 1 MHz to 1 mHz with a perturbation amplitude of ± 10 mV using VMP3 (Bio‐Logic). The full cell was prepared with SPAN powder as the cathode and K–metal as the anode. The cathode was prepared by casting a slurry containing the active materials, carbon black (Super P), and PAA binder (8:1:1 by weight percent) onto an Al foil–coated Cu current collector. The average loading of the active materials was ≈1.8–2.0 mg cm^−2^. The full cell for the electrochemical measurements employed 2032 coin cells. The full cell was typically cycled in constant‐current mode at 4 mA cm^−2^ and 1 mAh cm^−2^ at 1.0–3.0 V.

### Computational Methods

Theoretical studies to calculate HOMO–LUMO energy level and solvation energy (*E*_solv_) were performed using the Vienna ab initio simulation (VASP) package.^[^ ^71^ ^]^ The generalized gradient approximation (GGA) with Perdew–Burke–Ernzerhof (PBE) exchange‐correlation functional was employed to optimize molecule geometry.^[^ ^72^ ^]^ The wavefunction cutoff energy was set to 400 eV with the energy and force convergence threshold criteria of 10^−6^ eV and 0.02 eV Å^−1^, respectively. The molecules were added in a cubic box with a size of 15 × 15 × 15 Å^3^. For *E*_solv_ calculation, an implicit solvation model was employed and implemented in the VASPsol module developed by the Hennig group.^[^ ^73^ ^]^ The solvent dielectric constant was set to 7.2 for the DME. The *E*_solv_ was calculated using the following formula equation:

(5) $$E_{\mathrm{solv}}=E_{\mathrm{tot};\mathrm{sol}}--E_{\mathrm{tot};\mathrm{vac}}$$

where *E*_tot;sol_ and *E*_tot;vac_ are the total energies of the system under vacuum and solvent environments, respectively. To visualize molecule models, the VESTA program was used.^[^ ^74^ ^]^ Molecular dynamics simulation was carried out by GROMACS 2020.2 software. The number of molecule 100 KFSI/900 DME and 100 KFSI/900 DME + 5 KPF_6_ for 1 M KFSI/DME and 1 M KFSI/DME + 0.05 M KPF_6_ respectively. A 7.5 × 7.5 × 7.5 nm cubic box was created with a total volume of 421.875 nm for each system. OPLS force field was used for all‐atom accurate calculation with verlet integration to accurately calculate trajectories of particles in molecular dynamics simulation as shown in equations.

(6) $$\vec{x}\left(t+\Delta t\right)=\vec{x}\left(t\right)+\vec{v}\left(t\right)\Delta t+\frac{1}{2}\vec{a}\left(t\right)\Delta t^{2}$$

(7) $$\vec{x}\left(t+\Delta t\right)=\vec{v}\left(t\right)+\frac{\vec{a}\left(t\right)+\vec{a}\left(t+\Delta t\right)}{2}\Delta t$$

Minimization energy was carried out for 1 ns with the steepest descent minimization. 10 ns NPT ensemble followed by 10 ns NVT ensemble were carried out at a constant number of atoms in the system, pressure (NPT), volume (NVT), and temperature with Particle mesh Ewald Coulomb‐type for long‐range electrostatic calculation and cubic interpolation, V‐rescale temperature coupling with modified Berendsen thermostat were applied at temperature 298 K and 1 atm at isotropic periodic box condition (PBC) box vectors. Finally, 20 ns MD production was carried out to define the molecular interaction and condition during simulation.

### Characterization Methods

The SEI layer formed on the surface of free‐standing CNT film after was observed by HR‐TEM (Cs‐corrected TEM with Cold FEG, JEM ARM200F JEOL Ltd). The SEI layer on the surface of the CNT film was prepared using a holey carbon grid. TEM samples were loaded under temperature‐controlled conditions to prevent electron beam contamination (−110 °C). The solvation structure of the electrolytes and the bonding structure of SPAN were analyzed using a laser Raman spectrophotometer (Raman, NRS–5100). The morphology of the deposited K used separators after cycling, and constituents of SPAN was confirmed by field‐emission scanning electron microscopy (FE‐SEM, HITACHI S–4700 instruments). AFM characterizations were conducted on a high‐resolution AFM (HR‐AFM) scanning probe microscope (probes), unless otherwise indicated, in an Ar‐filled glove box. Non‐contact mode images of the surface morphology were obtained using silicon AFM tips coated Al with a typical scanning rate of 0.5 Hz. Force curves of the samples were obtained by force spectroscopy mode using the silicon probes coated diamond with a spring constant of 7.2 N m^−1^ subject to the hardness of the samples. Typically, probes with a radius of 10 nm were used for both samples. The thickness and Young's modulus of SEIs were estimated based on data collected at the topography image. High‐performance X‐ray photoelectron spectroscopy (HP‐XPS, K– LPHA+) was used to evaluate the components of the SEI layer formed on the surface of the K foil. Raman and FTIR (Spectrum 400) were also used to compare the bonding structure of SPAN before and after cycling. Operando OM was performed using customized visualization cell parts for collecting the electrochemical as well as morphological information simultaneously. Cell assembly was conducted in a glove box filled with Ar gas to prevent degradation to K‐metal and the electrolyte. A quartz observation window was installed to allow the transmission of light to the metal surfaces. Operando OM measurements were performed using Ar‐filled customized cells by employing a potentiostat (Bio‐Logic, VMP3), over the voltage range of 5 to −5 V, at a current density of 8 mA. Ultra‐high accuracy OM (VHX–7000) was used to acquire videos.

## Conflict of Interest

The authors declare no conflict of interest.

## Supporting information

Supporting Information

Supplemental Movie 1

Supplemental Movie 2

## Data Availability

The data that support the findings of this study are available from the corresponding author upon reasonable request.
