# Supplementary material for: Regulating the Solvation Structure of Electrolyte via Dual–Salt Combination for Stable Potassium Metal Batteries
Source: Adv Sci (Weinh). 2023 Apr 17;10(16):2301201. doi: 10.1002/advs.202301201 (PMC10238220; doi:10.1002/advs.202301201)
Supplement: Supplementary file 1 — Supporting Information [file ADVS-10-2301201-s002.pdf]

Supporting Information

**Regulating the Solvation Structure of Electrolyte via Dual–Salt Combination for Stable Potassium Metal Batteries**

*Jimin Park, Gwangeon Oh, Un–Hyuck Kim, Muhammad Hilmy Alfaruqi, Xieyu Xu, Yangyang Liu, Shizhao Xiong, Adi Tiara Zikri, Yang–Kook Sun* Jaekook Kim* and Jang–Yeon Hwang**

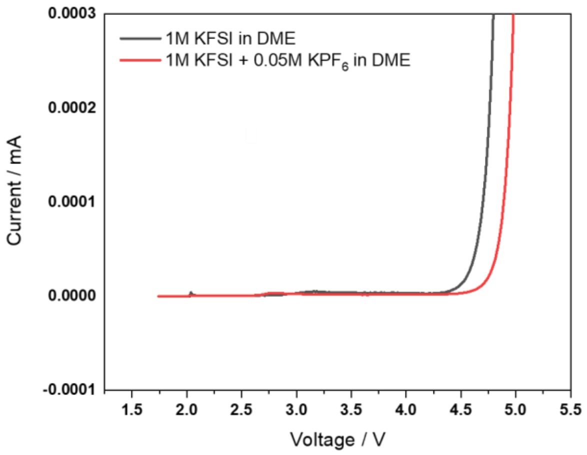


Figure S1. Linear sweep voltammetry data (scan rate: 0.1 mV $s^{-1}$) for the baseline (black line) and $KPF_6$ containing electrolyte (red line).

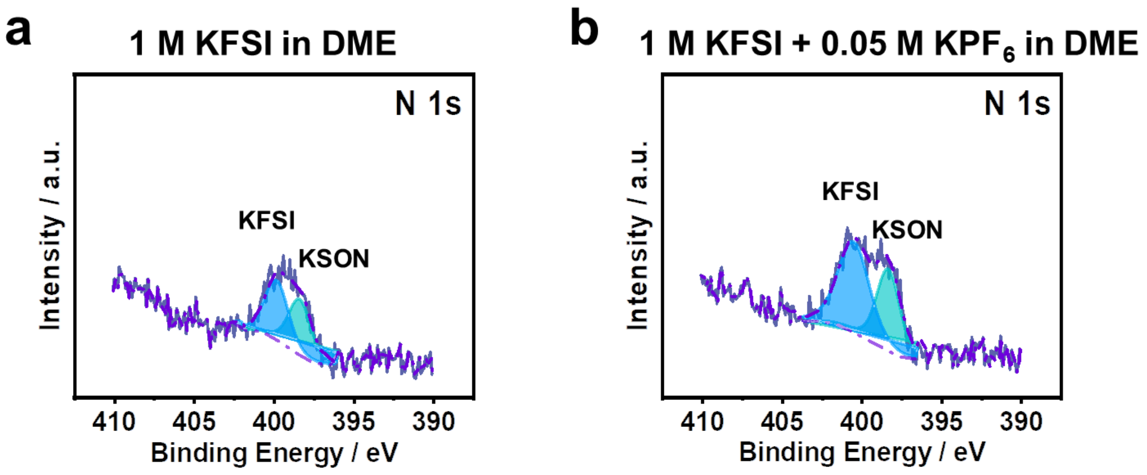


Figure S2. **XPS spectra of N 1s for SEI layer depending on different electrolyte solution: (a) 1 M KFSI in DME and (b) 1 M KFSI + 0.05 M $KPF_6$ in DME.**

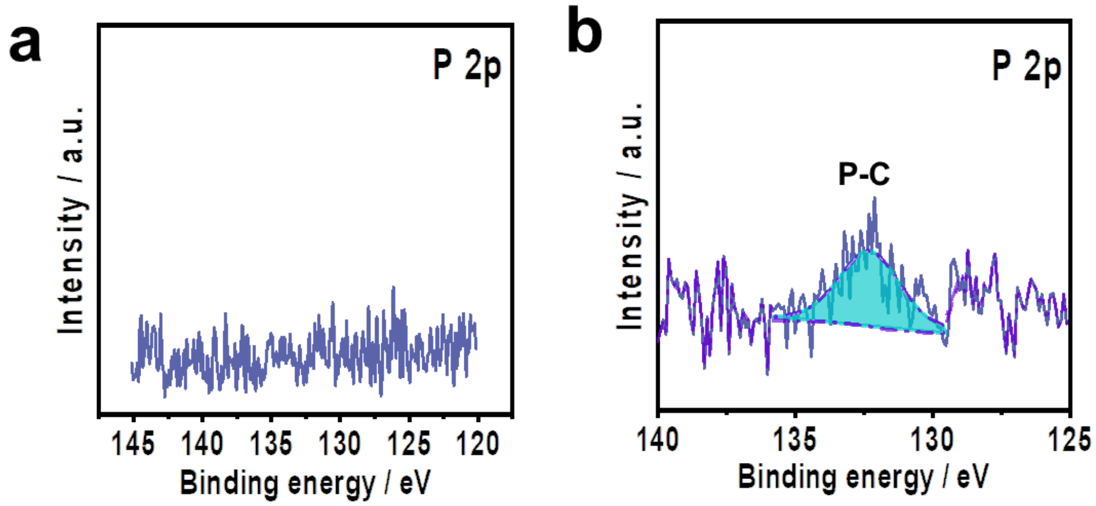


**Figure S3.** Characterization of the chemical component of SEI layers: XPS spectra of P 2p (a) baseline and 0.05 M $KPF_6$–containing electrolyte.

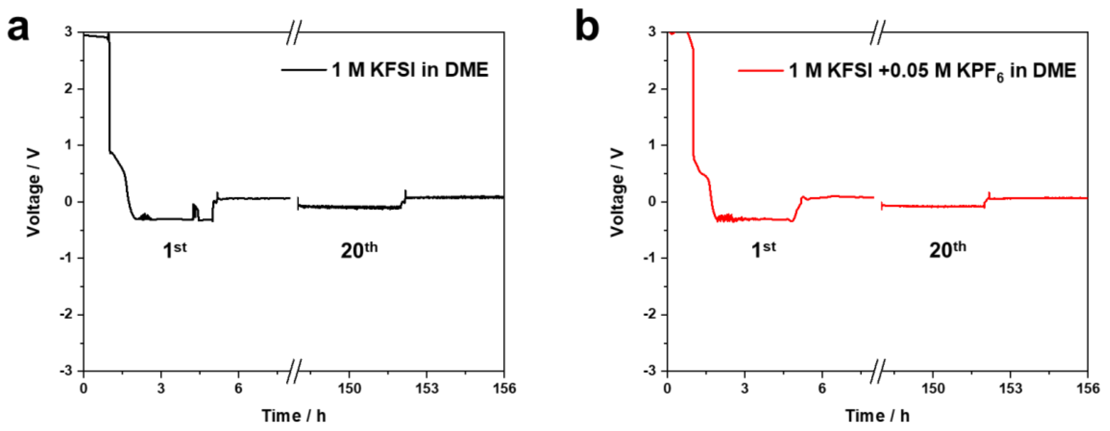


**Figure S4.** Galvanostatic plating/stripping test curves of K/CNT cells using different electrolyte at a current density of 1 mA $cm^{-2}$ and capacity loading of 4 mAh $cm^{-2}$ during 20 cycles: (a) baseline electrolyte and (b) 0.05 M $KPF_6$ –containing electrolyte.

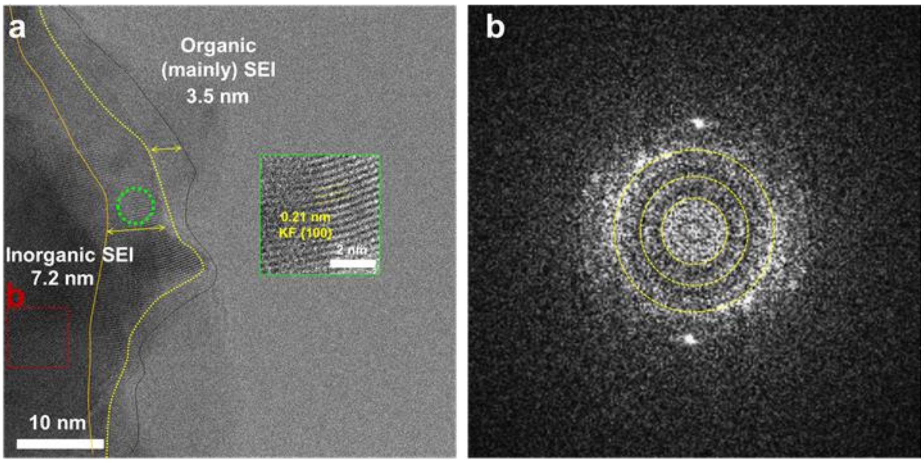


**Figure S5.** (a) Low-temperature HR–TEM image of SEI layer on CNT surface cycled in the $KPF_6$ containing electrolyte and (b) FFT data collected from red square region in Figure S5a.

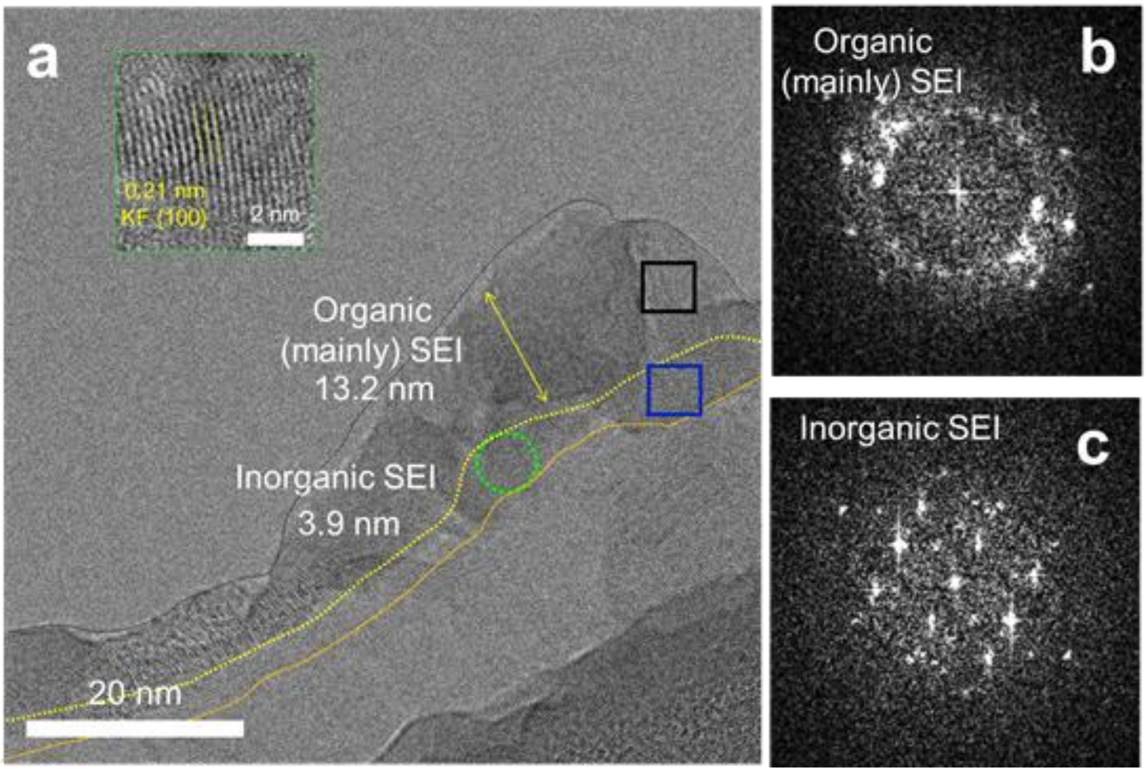


**Figure S6.** (a) Low-temperature HR–TEM image of SEI layer on CNT surface cycled in the baseline electrolyte and FFT data collected from (b) black and (c) blue square region, respectively.

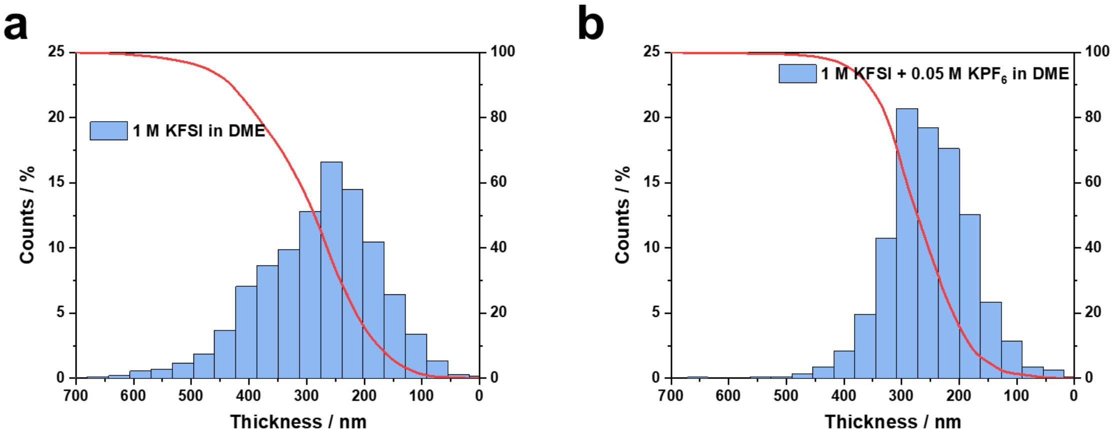


**Figure S7.** The histograms of thickness corresponding to Fig. 3 (j and m); (a) baseline electrolyte and (b) 0.05 M $KPF_6$–containing electrolyte.

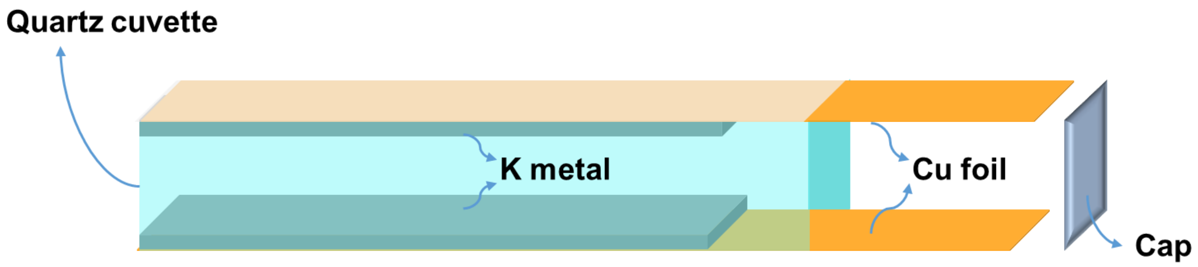


Figure S8. **Customized electrochemical cell for operando optical microscope for observing potassium electrodeposition.**

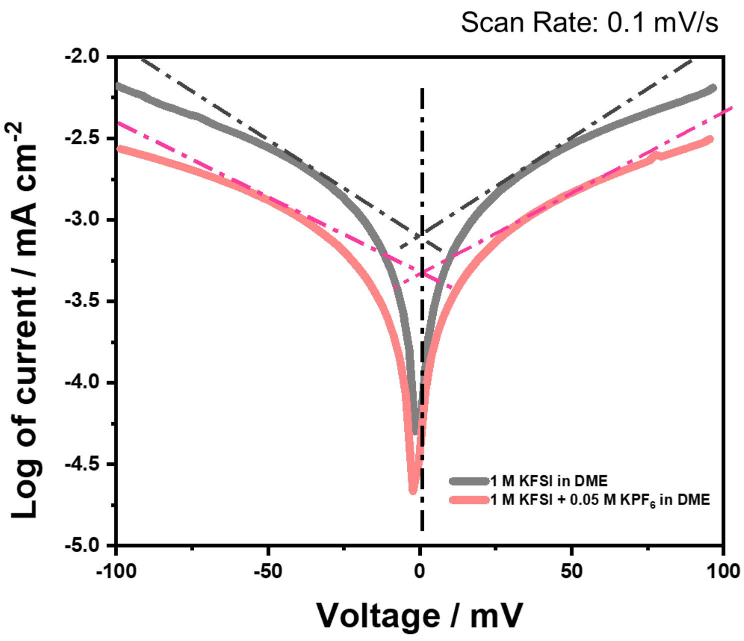


Figure S9. **Tafel plots of K–electrodes in different electrolyte solutions.**

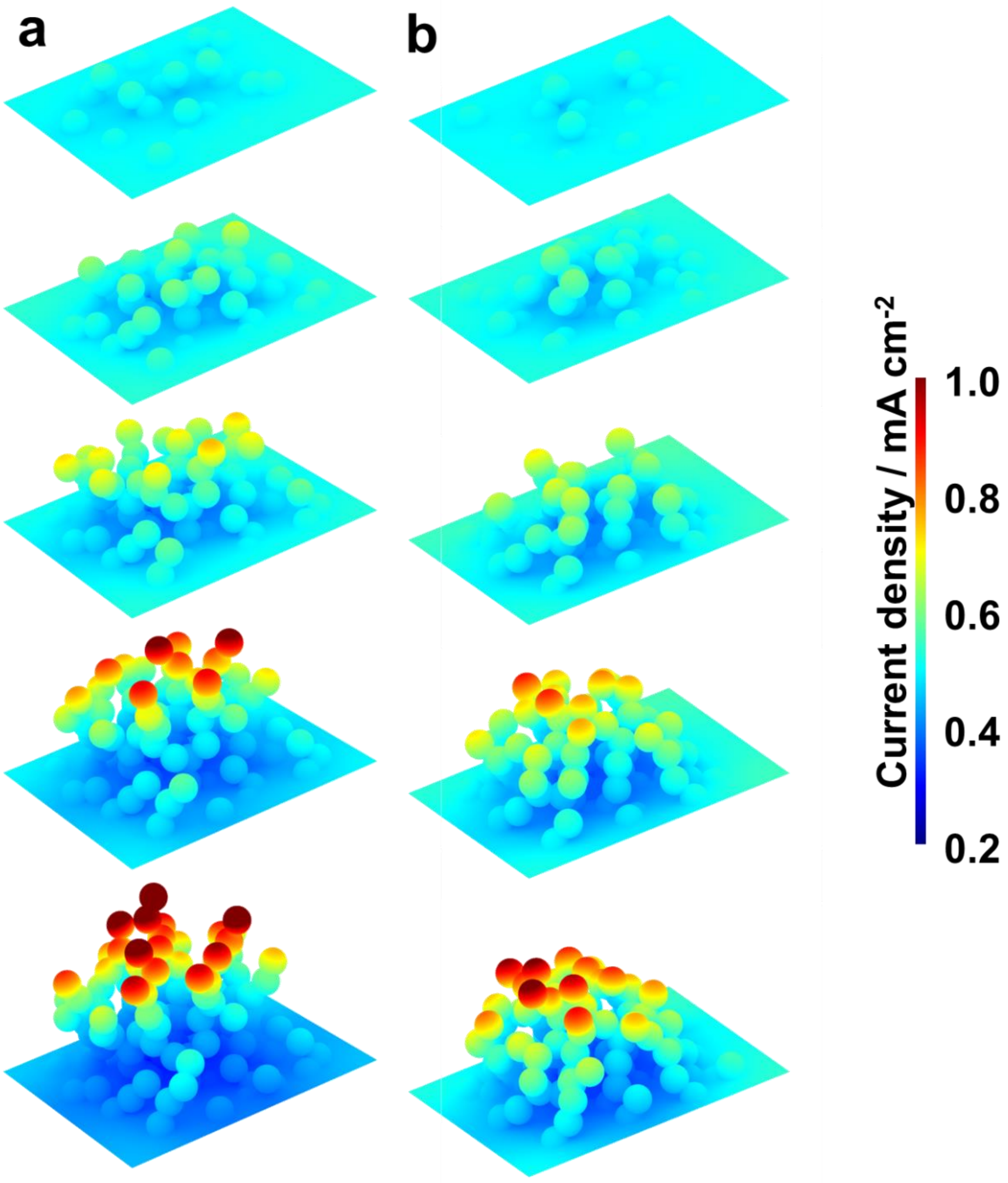


**Figure S10.** View (45°) of simulated morphological evolution of K–metal using electrolyte comprising (a) baseline electrolyte and (b) 0.05 M $KPF_6$–containing electrolyte.

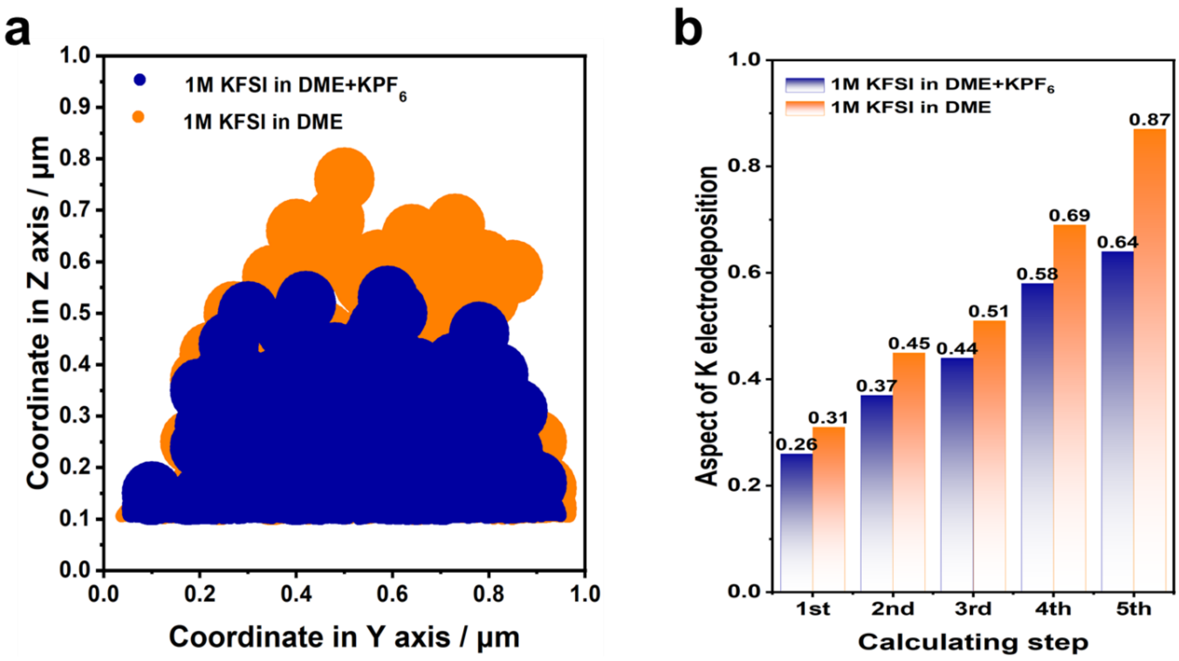


Figure S11. (a) Cross–section showing simulated morphology evolution of K–metal with electrolyte comprising baseline electrolyte and 0.05 M $KPF_6$–containing electrolyte. (b) Aspect changes of deposited K during successive electrodeposition process.

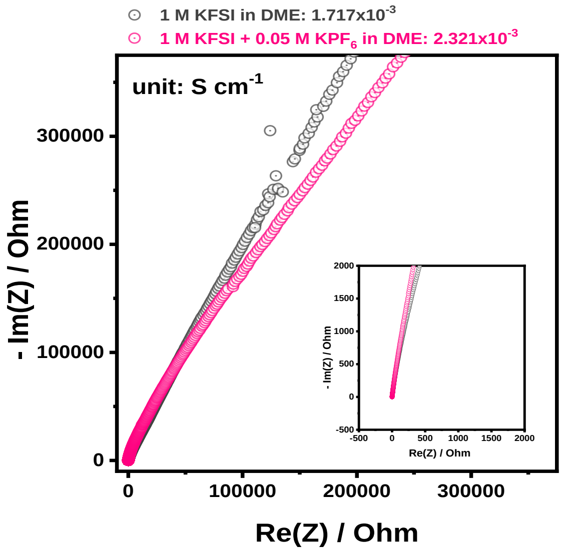


**Figure S12.** Impedance plots of electrolyte with/without $KPF_6$ salt. The ionic conductivity of 0.05 M $KPF_6$ –containing electrolyte was calculated to be 2.321 x $10^{-3}$ S $cm^{-1}$ at room temperature, which was higher than that of baseline electrolyte (1.717 x $10^{-3}$ S $cm^{-1}$).

The ionic conductivity was conducted by symmetric cells using stainless steel electrodes. The electrolyte impedance was evaluated by EIS. Ionic conductivity was calculated according to the following equation,

$$\sigma = \frac{d}{RA} \cdots\cdots (1)$$

where **σ** is the ionic conductivity (S $cm^{-1}$), d is the thickness of the separa**tor (cm), R is the resistance (Ω)**, and the area of the stainless steel electrode ($cm^2$).

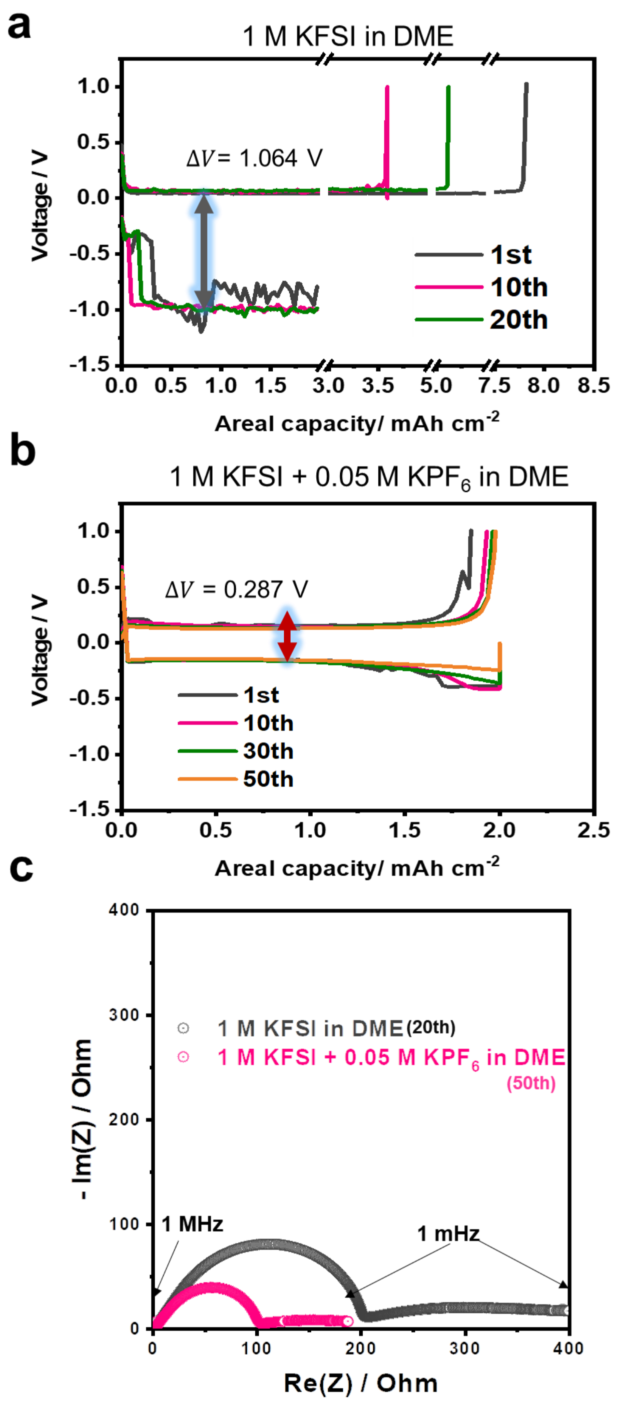


**Figure S13.** Voltage profiles of asymmetric K | Cu cells using different electrolyte solutions at a current density of 1 mA cm$^{-2}$ and capacity loading of 1 mAh cm$^{-2}$: (a) baseline electrolyte and (b) 0.05 M $KPF_6$–containing electrolyte. (c) AC impedance spectra corresponding to K | Cu asymmetric cells after 20th (baseline) and 50th (with 0.05 M $KPF_6$) cycle, respectively.

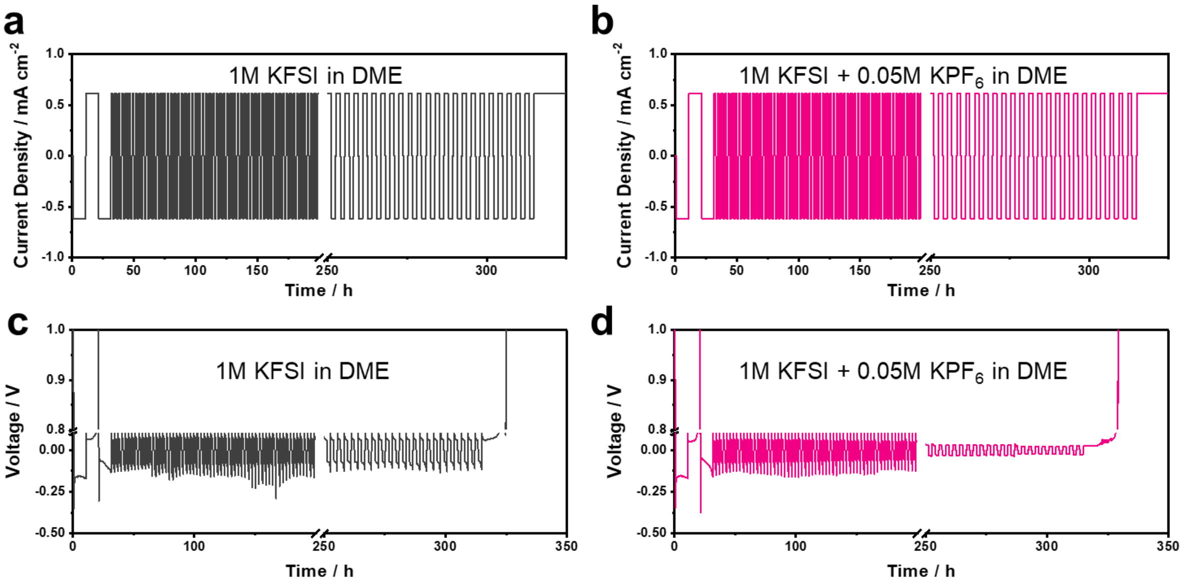


**Figure S14.** Measurement of average coulombic efficiency of K–metal anode depending on different electrolyte solutions. (a and b) Constant–current protocol and (c and d) measured voltage versus time plot for K | Cu cells.

Owing to the high sensitivity of electrodeposit K against the Cu substrate, it is generally suggested that the modified K/Cu test protocol suggested by the Zhang et al.[1] to check the fundamental effect of electrolyte solution on SEI formation and/or interfacial properties on K metal anode surface. In this method, a given amount of charge ($Q_T$) is used to deposit K onto the Cu substrate first as a K reservoir, then a smaller portion of this charge ($Q_c$) is used to cycle K between working and counter electrodes for $n$ cycles. After $n$ cycles, a final exhaustive strip of the remaining K reservoir is performed to the cut–off voltage. The final stripping charge ($Q_S$), corresponding to the quantity of K remaining after cycling, is measured.

[1] B. D. Adams, J. Zheng, X. Ren, W. Xu, J.–G. Zhang, *Adv. Energy Mater.* **2018,** *8*, 1702097.

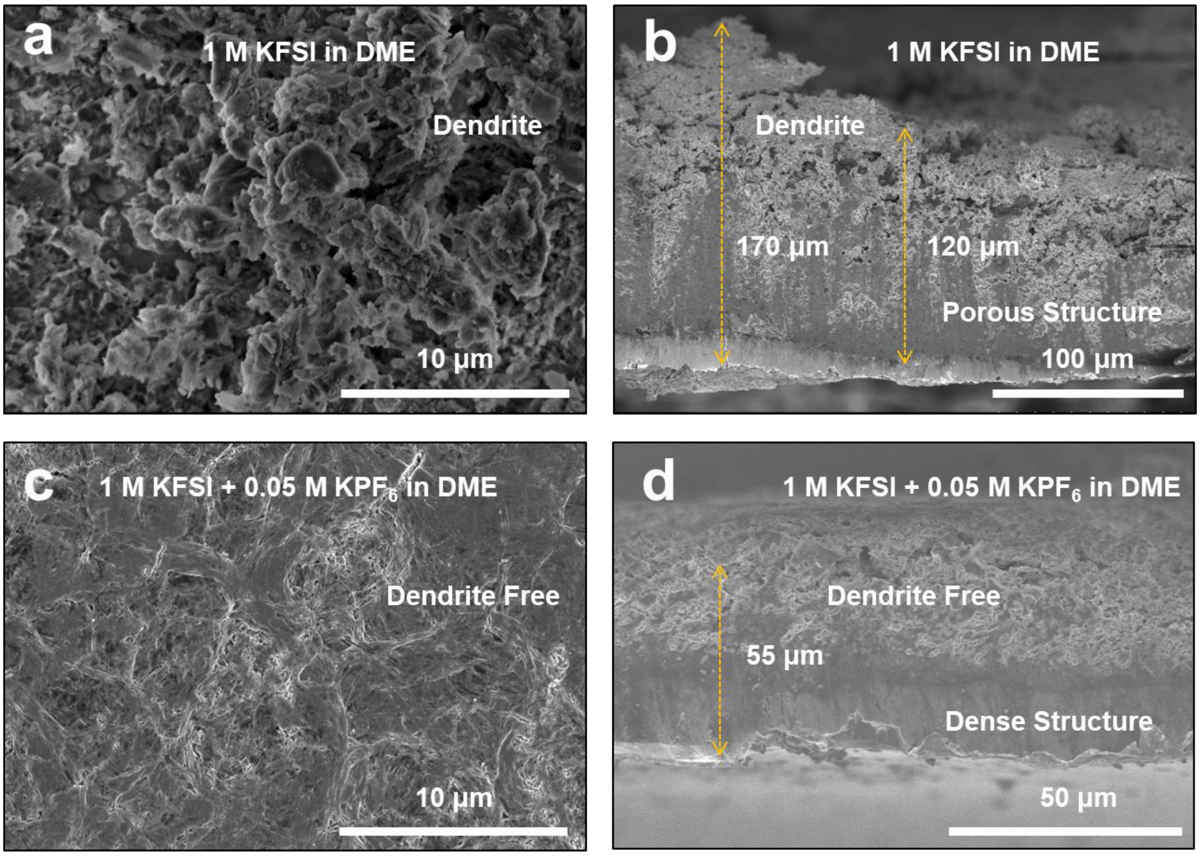


**Figure S15.** Top–view and cross–sectional SEM images of accumulated K–metal on Cu substrate obtained from asymmetric K | Cu cells employing different electrolyte solutions: (a and b) baseline electrolyte (after 20 cycles) and (c and d) 0.05 M $KPF_6$–containing electrolyte (after 50 cycles).

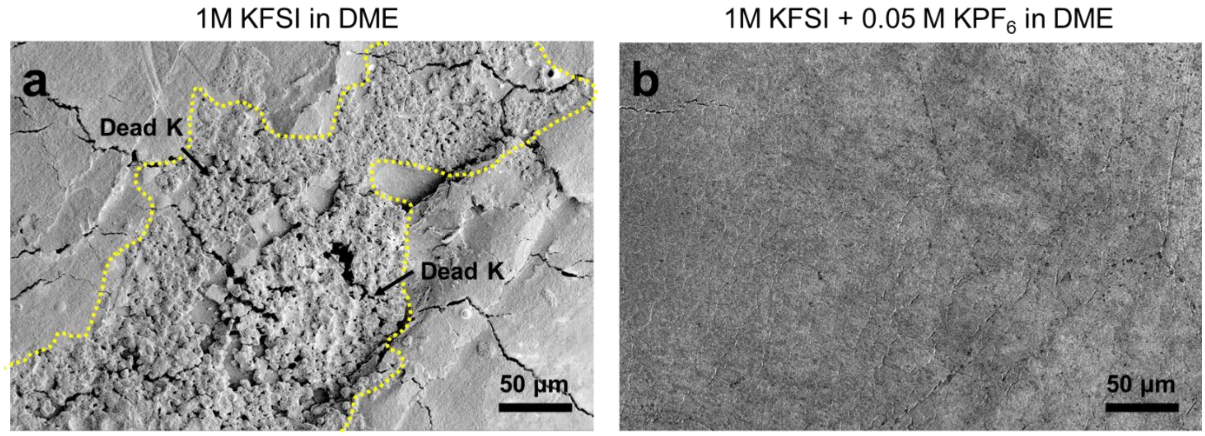


Figure S16. Top-view of SEM images of the Cu foil after stripping process (10 cycles) at 1 mA cm$^{-2}$ and 1 mAh cm$^{-2}$ (a) baseline electrolyte and (b) 0.05 M $KPF_6$–containing electrolyte.

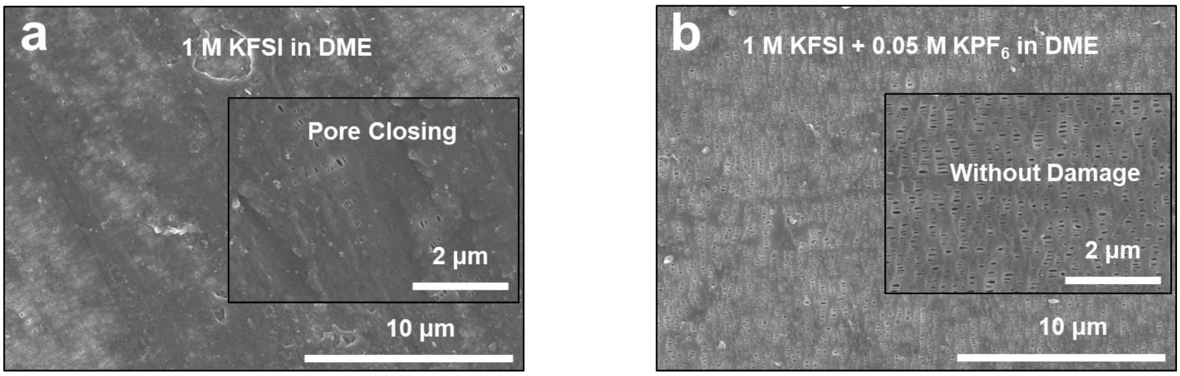


**Figure S17.** SEM images of cycled separator collected from asymmetric K | Cu cells with different electrolyte solutions: (a) baseline electrolyte (after 20 cycles) and (b) 0.05 M $KPF_6$–containing electrolyte (after 50 cycles).

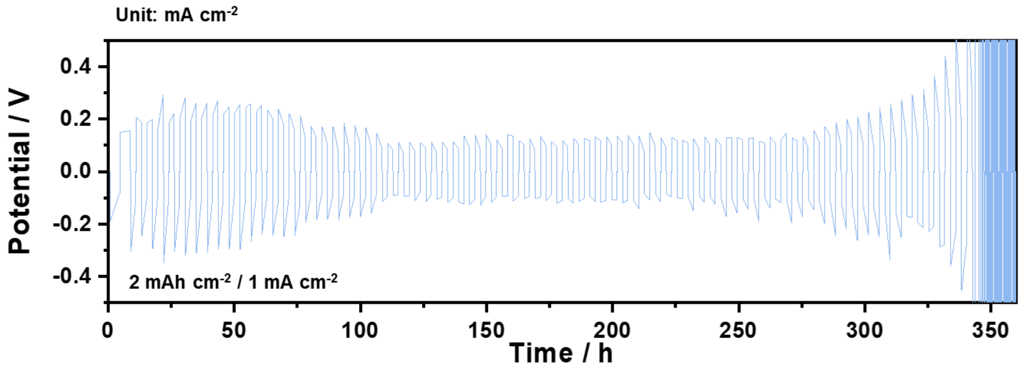


**Figure S18.** Galvanostatic cycling test of K | K symmetric cells with 0.1 M $KPF_6$–containing electrolyte cycled at a current density of 1 mA cm$^{-2}$ and capacity loading of 2 mAh cm$^{-2}$ with electrolyte amount of 100 $\mu$l.

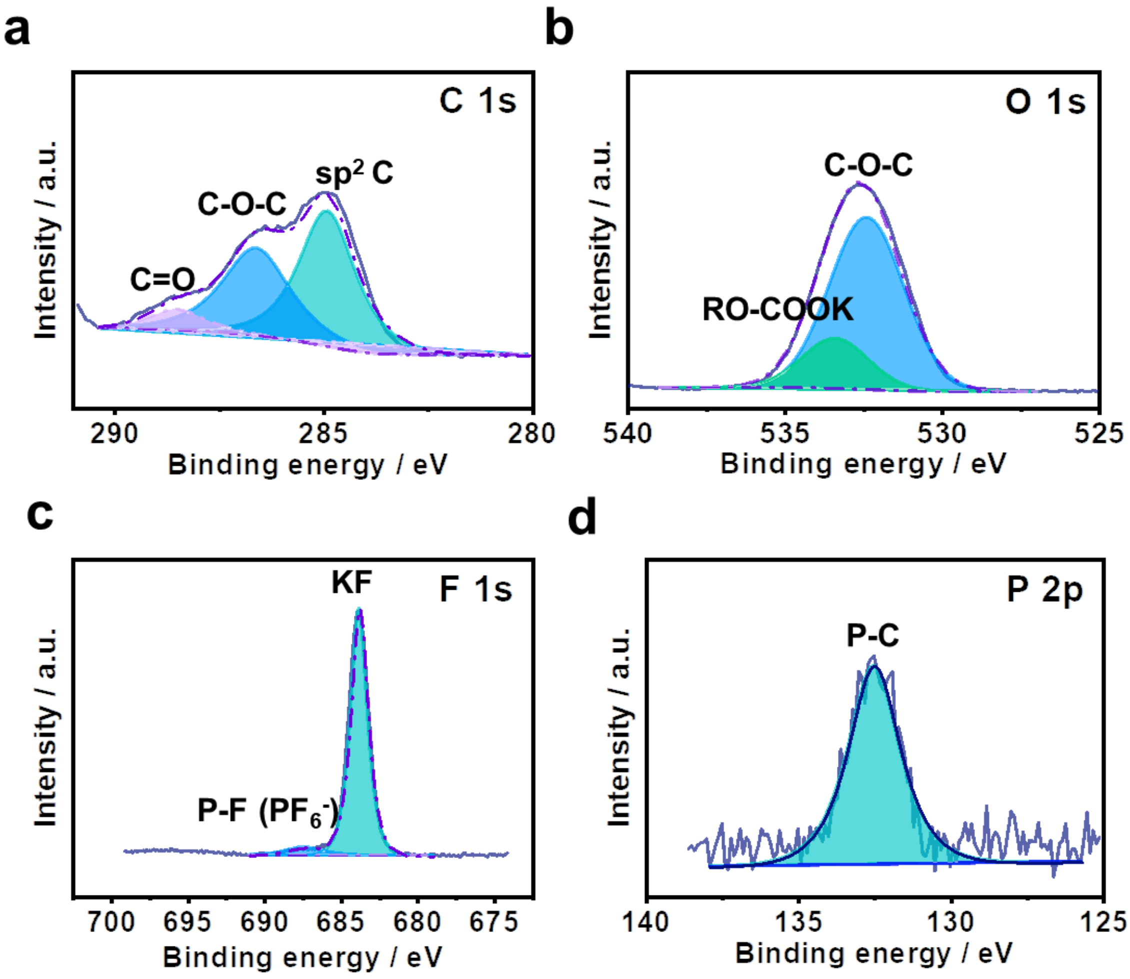


**Figure S19.** XPS spectra of the chemical component of SEI layers of 0.1 M $KPF_6$–containing electrolyte. XPS spectra of C 1s, O 1s, F 1s and P 2p.

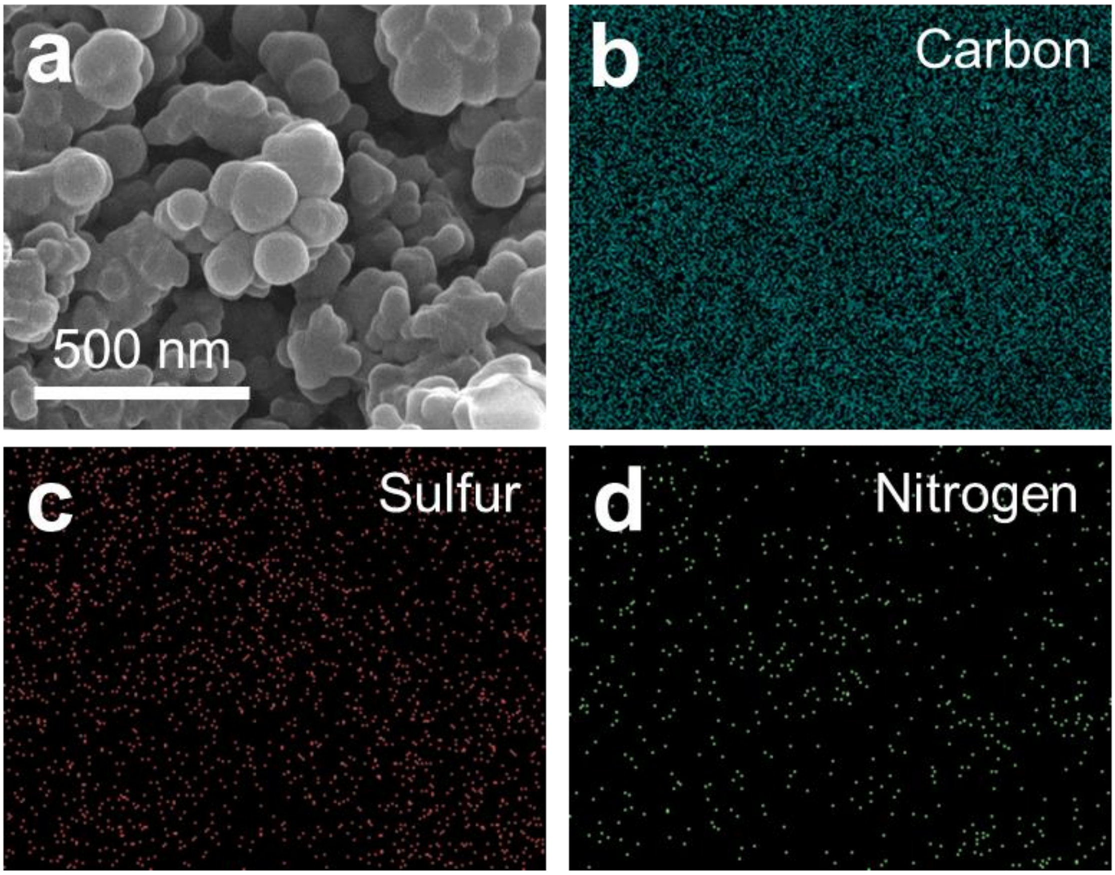


**Figure S20.** SEM Images of (a) SPAN and (b–d) corresponding elemental maps of C, S, and N.

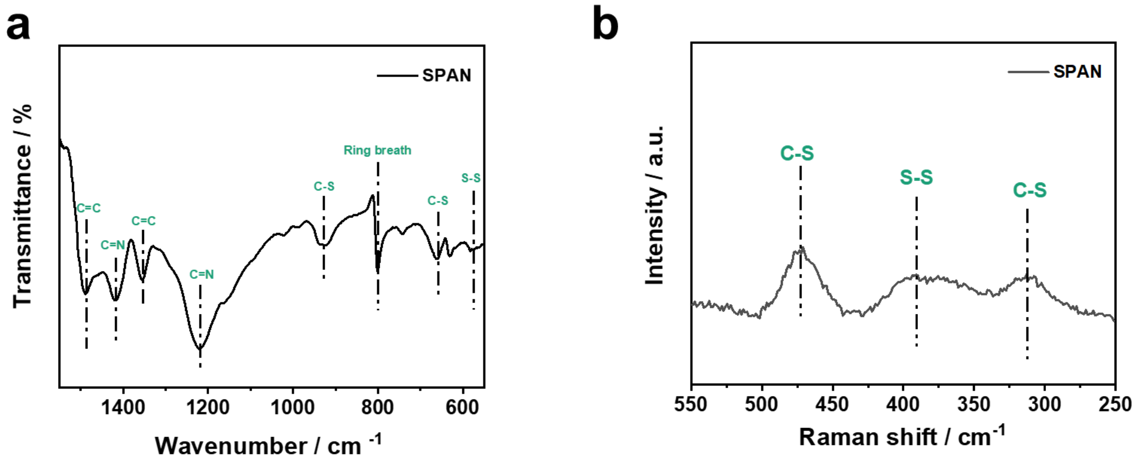


**Figure S21.** (a) FTIR and (b) Raman spectra of SPAN.

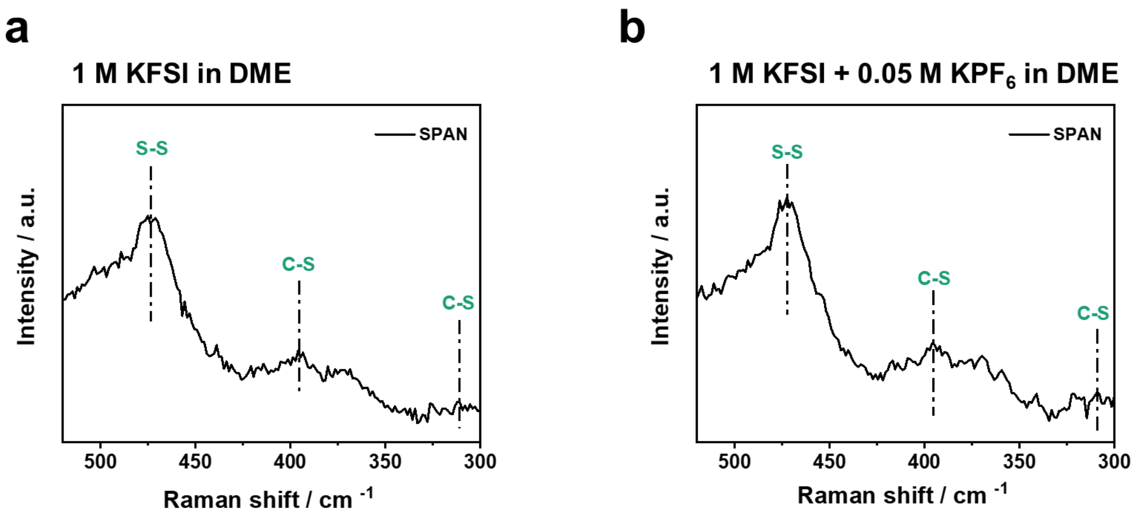


**Figure S22.** Raman spectra of cycled SPAN cathodes (after 300 cycles) with (a) baseline electrolyte and (b) 0.05 M $KPF_6$–containing electrolyte.

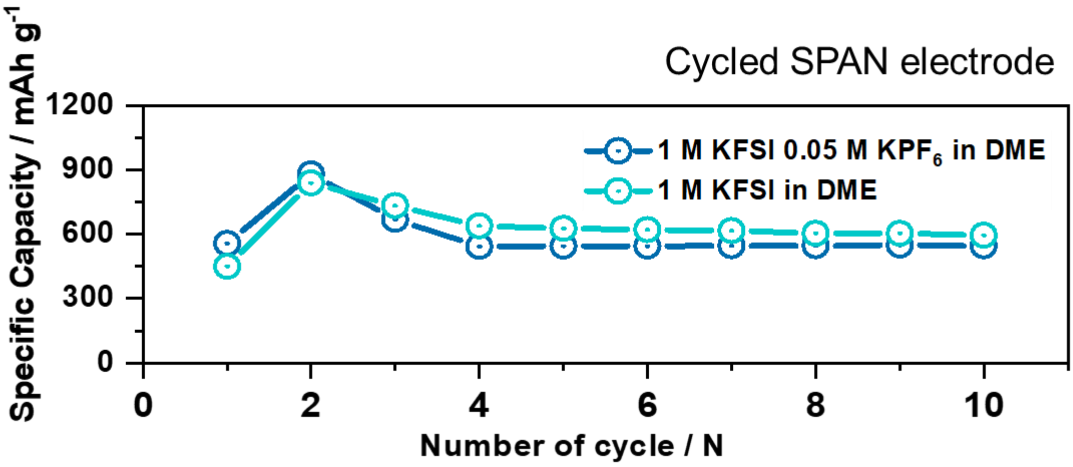


**Figure S23.** Electrochemical performance of K–S batteries using cycled SPAN cathodes (after 300 cycles) with fresh electrolyte solution: (a) baseline electrolyte and (b) 0.05 M $KPF_6$–containing electrolyte.

**Table S1.** K–Metal stability in 1 M KFSI + 0.05 M $KPF_6$ in DME electrolyte compared with previous literatures.

| Electrolyte composition | Current density ( mA $cm^{-2}$)<br>&<br>Capacity ( mAh $cm^{-2}$) | Electrolyte amount (µL) | Separator | Cycle time (h) | Overpotential (V) | Ref. |
|---|---|---|---|---|---|---|
| 1 M KFSI + 0.05 M $KPF_6$ in DME | a. 1 mA $cm^{-2}$/1 mAh $cm^{-2}$<br>b.4 mA $cm^{-2}$/1 mAh $cm^{-2}$<br>c. Rate test/1 mAh $cm^{-2}$<br>(1 mA $cm^{-2}$ to 10 mA $cm^{-2}$)<br>d. 1 mA $cm^{-2}$/1 mAh $cm^{-2}$ | a–c. 100 µL<br>d. 50 µL | Celgard®2400 (Celgard, LLC Corp.) | a. 1100 h<br>b. 410 h<br>c. 500 h<br>d. 600 h | a. 0.5 V<br>b. 0.5 V<br>c. 1 V at 10 mA $cm^{-2}$<br>d. 0.4 V | This work |
| a. 0.8 M $KPF_6$ in EC:DEC (1:1, v/v)<br>b. 0.8 M $KPF_6$ in EC:DEC (1:1, v/v) + 1 vol % FEC<br>c. 0.8 M $KPF_6$ in EC:DEC (1:1, v/v) + 1 vol % VC<br>d. 0.8 M $KPF_6$ in EC:DEC (1:1, v/v) + 1 vol % DFEC<br>e. 0.8 M $KPF_6$ in EC:DEC (1:1, v/v) + 1 vol % ES<br>f. 0.8 M $KPF_6$ in EC:DEC (1:1, v/v) + 1 wt % TMS<br>g. 0.8 M $KPF_6$ in EC:DEC (1:1, v/v) + 1 wt % DTD | 25 $\mu$A $cm^{-2}$/250 $\mu$Ah $cm^{-2}$ | N/A | Glass fiber separator (GB–100R, Advantec) | 250 h | a. 0.4V<br>b,d. 1 V<br>c. 0.2 V<br>e,f. 0.25 V | [1] |
| a. 0.8 M $KPF_6$ in EC:DEC (1:1, v/v)<br>b. 1 M KFSI in EC:DEC (1:1, v/v)<br>c. 2 M KFSI in TEP (1:1, v/v) | 1 mA $cm^{-2}$/1 mAh $cm^{-2}$ | N/A | Glass fiber | a. 360 h<br>b. 750 h<br>c. 750 h | a. Unstable<br>b. 500 mV<br>c. 428 mV to 137 mV | [2] |
| a. 2 M KFSI in EC:DEC (1:1, v/v)<br>b. 1 M KFSI in EC:DEC (1:1, v/v)<br>c. 0.5 M KFSI in EC:DEC (1:1, v/v)<br>d. 0.25 M KFSI in EC:DEC (1:1, v/v) | 0.5 mA $cm^{-2}$/0.5 mAh $cm^{-2}$ after pre–cycling at 0.1 mAh $cm^{-2}$/0.5 mAh $cm^{-2}$ | 80 µL | Celgard–2320 (polypropylene polyethylenepolypropylene) and glass fiber (Whatman, GF/D) | a. short circuit at pre–cycling<br>b. 290 h<br>c. 400 h<br>d. 160 h | a. 0.5 V<br>b. 1 V<br>c. 1 V<br>d. 0.5 V | [3] |

| | | | | | | |
|---|---|---|---|---|---|---|
| 0.8 M $KPF_6$ in EC:DEC:PC (2:1:2, v/v/v) | a. 0.5 mA $cm^{-2}$/0.5 mAh $cm^{-2}$<br>b. Rate test/0.5 mAh $cm^{-2}$<br>(0.1 mA $cm^{-2}$ to 2 mA $cm^{-2}$) | 120 μL | Glass microfiber (Whatman) | a. 86 h<br>b. 51 h | a. ~0.5 V<br>b. 0.5 V at 0.1 mA $cm^{-2}$ | [4] |
| a. 0.8 M $KPF_6$ in EC:DEC (1:1, v/v)<br>b. 1 M KFSI in EC:DEC (1:1, v/v) | 1 mA $cm^{-2}$/1 mAh $cm^{-2}$<br>Rate test/1 mAh $cm^{-2}$<br>(1 mA $cm^{-2}$ to 5 mA $cm^{-2}$) | N/A | Glass fiber | a. 24 h/37 h<br>b. 200 h/70 h | a. 0.6 V at 2 mA $cm^{-2}$<br>b. 0.8 V at 5 mA $cm^{-2}$ | [5] |
| a. 2.3 M KFSI in DME<br>b. 2.3 M KFSI + 50 Mm $KNO_3$ in DME | a. 0.05 mA $cm^{-2}$/0.15 mAh $cm^{-2}$<br>b. 0.25 mA $cm^{-2}$/0.25 mAh $cm^{-2}$<br>c. 1mA $cm^{-2}$/0.25 mAh $cm^{-2}$ | N/A | Polypropylene (PP, Celgard 2400) and glass fiber (GF/A, Whatman) separators, with a PP/GF/PP configuration | a. 300 h<br>b. 200 h<br>c. 100/200 h | a. 0.4 V<br>b. 0.8 / 0.4 V<br>c. 1.2 / 0.6 V | [6] |
| a. 0.8 M $KPF_6$ in EC:DEC (1:1, v/v)<br>b. 1 M KFSI in EC:DEC (1:1, v/v)<br>c. 0.8 M $KPF_6$ in EC:DEC (1:1, v/v) + 5 wt.% FEC<br>d. 1 M KFSI in EC:DEC (1:1, v/v) + 5wt.% FEC | 1 mA $cm^{-2}$/1 mAh $cm^{-2}$ | N/A | N/A | a. 42 h<br>b. 208 h<br>c. 97 h<br>d. 208 h | a,b. ±200 mV<br>c,d. ±500 mV | [7] |
| a. 0.5 M $KPF_6$ in EC:DCE<br>b. 0.5 M $KPF_6$ in EC:DEC + 0.1 wt.% KDFP<br>c. 0.5 M $KPF_6$ in EC:DEC + 0.2 wt.% KDFP | Rate test<br>10 μA $cm^{-2}$/1.3 μAh $cm^{-2}$<br>50 μA $cm^{-2}$/6.6 μAh $cm^{-2}$<br>100 μA $cm^{-2}$/13.3 μAh $cm^{-2}$<br>500 μA $cm^{-2}$/66.6 μAh $cm^{-2}$ | 100 μL | Glass fiber filter (Whatman, GF/A, 260 μm) | 13.3 h | 0.7 V at 500 μA $cm^{-2}$ | [8] |
| a. 1 M KFSI in EC:DEC (1:1, v/v)<br>b. 1 M KFSI in DME<br>c. 4 M KFSI in DME | 1 mA $cm^{-2}$/1 mAh $cm^{-2}$ | N/A | Glass fiber membrane | a. 320 h<br>b. 430 h<br>c. 600 h | a. 0.6 V<br>b. 0.3 V<br>c. 0.04 V | [9] |
| 1 M KFSI in DME | 0.2 mA $cm^{-2}$/0.2 mAh $cm^{-2}$ | N/A | Glass fiber | < 50 h | 0.05 V | [10] |
| a. 1 M KFSI in DME<br>b. 5 M KFSI in DME<br>c. 1 M KTFSI in DME<br>d. 5 M KTFSI in DME | 1 mA $cm^{-2}$/N/A | N/A | Trilayer polypropylene polyethylene membrane (Celgard 2325) | a. 50 h<br>b. 300 h<br>c. 50 h<br>d. 300 h | a. 1 V<br>b. 3─400 mV<br>c. 1 V<br>d. 500 mV | [11] |
| a. 1 M $KPF_6$ in DME<br>b. 1 M $KPF_6$ in | 0.1 mA $cm^{-2}$/0.1 mAh $cm^{-2}$ | 100 μL | Glass fiber | > 30 h | a. 10 mV<br>b. 600 mV | [12] |

| | | | | | | |
|---|---|---|---|---|---|---|
| PC | | | | | | |
| a. 0.8 M $KPF_6$ in EC:DEC (1:1, v/v)<br>b. 1 M KFSI in EC:DEC (1:1, v/v)<br>c. 3:8 (KFSI:TMP) | 1 mA $cm^{-2}$/1 mAh $cm^{-2}$ | N/A | Glass fiber | a. 110 h<br>b. > 500 h<br>c. more than 500 h | a. 300 mV<br>b. 600 mV<br>c. 200 mV | [13] |

**References in Table S1.**


[1] T. Hosaka, T. Fukabori, T. Matsuyama, R. Tatara, K. Kubota, S. Komaba, *ACS Energy Lett.* **2021**, *6*, 3643–3649.

[2] S. Liu, J. Mao, Q. Zhang, Z. Wang, W. K. Pang, L. Zhang, A. Du, V. Sencadas, W. Zhang, Z. Guo, *Angew. Chemie–Int. Ed.* **2020**, *59*, 3638–3644.

[3] Y. Gao, Z. Hou, R. Zhou, D. Wang, X. Guo, Y. Zhu, B. Zhang, *Adv. Funct. Mater.* **2022**, *32*, 2112399.

[4] P. Liu, Y. Wang, Q. Gu, J. Nanda, J. Watt, D. Mitlin, *Adv. Mater.* **2020**, *32*, 1906735.

[5] H. Wang, D. Yu, X. Wang, Z. Niu, M. Chen, L. Cheng, W. Zhou, L. Guo, *Angew. Chemie* **2019**, *131*, 16603–16607.

[6] H. Wang, J. Dong, Q. Guo, W. Xu, H. Zhang, K. C. Lau, Y. Wei, J. Hu, D. Zhai, F. Kang, *Energy Storage Mater.* **2021**, *42*, 526–532.

[7] W. Zhang, W. K. Pang, V. Sencadas, Z. Guo, *Joule* **2018**, *2*, 1534–1547.

[8] H. Yang, C.–Y. Chen, J. Hwang, K. Kubota, K. Matsumoto, R. Hagiwara, *ACS Appl. Mater. Interfaces* **2020**, *12*, 36168–36176.

[9] F. Yang, J. Hao, J. Long, S. Liu, T. Zheng, W. Lie, J. Chen, Z. Guo, *Adv. Energy Mater.* **2020**, *11*, 2003346.

[10] Q. Yang, Y. Ding, G. He, *Chem. Commun.* **2020**, *56*, 3512–3515.

[11] J. Touja, P. N. Le Pham, N. Louvain, L. Monconduit, L. Stievano, *Chem. Commun.* **2020**, *56*, 14673–14676.

[12] L. Zhou, Z. Cao, W. Wahyudi, J. Zhang, J.–Y. Hwang, Y. Cheng, L. Wang, L. Cavallo, T. Anthopoulos, Y.–K. Sun, H. N. Alshareef, J. Ming, *ACS Energy Lett.* **2020**, *5*, 766–776.

[13] S. Liu, J. Mao, L. Zhang, W. K. Pang, A. Du, Z. Guo, *Adv. Mater.* **2021**, *33*, 2006313.
